# Sand deposits reveal great earthquakes and tsunamis at Mexican Pacific Coast

**DOI:** 10.1038/s41598-020-68237-2

**Published:** 2020-07-10

**Authors:** María-Teresa Ramírez-Herrera, Néstor Corona, Jan Cerny, Rocío Castillo-Aja, Diego Melgar, Marcelo Lagos, Avto Goguitchaichvili, María Luisa Machain, Miriam L. Vazquez-Caamal, María Ortuño, Margarita Caballero, Ericka Alinne Solano-Hernandez, Ana-Carolina Ruiz-Fernández

**Affiliations:** 1 Laboratorio de Tsunamis y Paleosismología, Instituto de Geografía, Universidad Nacional Autónoma de México, Mexico, Mexico; 2 COLMICH, Centro de Estudios de Geografía Humana, Michoacán, México; 3 Laboratorio de Tsunamis y Paleosismología, UNAM, Mexico, México; 4 Depto. de Geografía y Ord. Territorial, CUCSH. Universidad de Guadalajara, Mexico, México; 5 Department of Earth Sciences, University of Oregon, Oregon, USA; 6 Instituto de Geografía, Laboratorio de Tsunamis, Pontificia Universidad Católica de Chile, Santiago, Chile; 7 Instituto de Geofísica, Universidad Nacional Autónoma de México, Unidad Michoacán, Mexico, México; 8 Instituto de Ciencias del Mar y Limnología, Universidad Nacional Autónoma de México, Mexico, Mexico; 9 Universidad del Mar, Ciudad Universitaria, Puerto Ángel, San Pedro Pochutla, Oax. Mexico; 10 Ciencias de La Tierra, Universidad de Barcelona, Barcelona, Spain

**Keywords:** Geology, Geomorphology, Seismology, Tectonics, Natural hazards, Solid Earth sciences

## Abstract

Globally, instrumentally based assessments of tsunamigenic potential of subduction zones have underestimated the magnitude and frequency of great events because of their short time record. Historical and sediment records of large earthquakes and tsunamis have expanded the temporal data and estimated size of these events. Instrumental records suggests that the Mexican Subduction earthquakes produce relatively small tsunamis, however historical records and now geologic evidence suggest that great earthquakes and tsunamis have whipped the Pacific coast of Mexico in the past. The sediment marks of centuries old-tsunamis validate historical records and indicate that large tsunamigenic earthquakes have shaken the Guerrero-Oaxaca region in southern Mexico and had an impact on a bigger stretch of the coast than previously suspected. We present the first geologic evidence of great tsunamis near the trench of a subduction zone previously underestimated as potential source for great earthquakes and tsunamis. Two sandy tsunami deposits extend over 1.5 km inland of the coast. The youngest tsunami deposit is associated with the 1787 great earthquake, M 8.6, producing a giant tsunami that poured over the coast flooding 500 km alongshore the Mexican Pacific coast and up to 6 km inland. The oldest event from a less historically documented event occurred in 1537. The 1787 earthquake, and tsunami and a probable predecessor in 1537, suggest a plausible recurrence interval of 250 years. We prove that the common believe that great tsunamis do not occur on the Mexican Pacific coast cannot be sustained.

## Introduction

Worldwide, instrumentally based assessments of tsunamigenic potential of subduction zones have underestimated the magnitude and frequency of great events^1–3^, to some extent because great earthquakes and tsunamis are infrequent^1–5^ and instrumental seismic data are relatively short. Historical and sediment records of large earthquakes and tsunamis have expanded the temporal data and estimated size of these events but mainly where direct observations of great tsunamis have been possible^1,3,4,6^. This means that little is still known of great earthquakes and tsunami generation potential of other subduction zones^7–11^. Additionally, at the centre of the problem is still the question as to whether subduction zones, despite their relatively short instrumentally seismic history, could generate great earthquakes and tsunamis. We reveal the first geologic evidence, and validate historical records, of great tsunamis and earthquakes near the trench of the Mexican subduction zone previously underestimated as potential source for great earthquakes and tsunamis.

**Figure 1:**
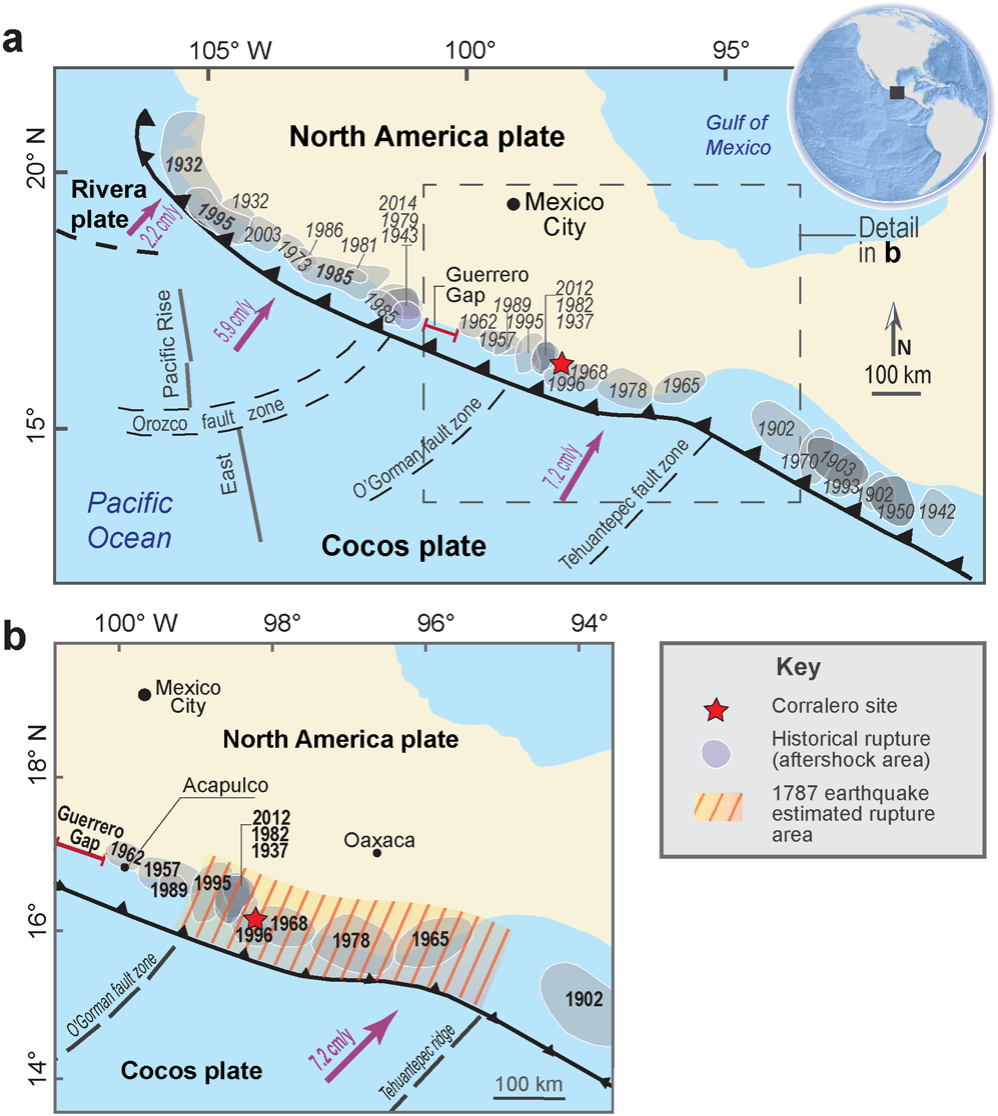
Maps of study area. Middle America trench and seaward edges of Mexican subduction zone are shown with barbed lines. (**a**) Mexican subduction zone showing historical rupture (aftershock) areas of the twentieth and twenty-first century earthquakes (modified after https://usuarios.geofisica.unam.mx/vladimir/images/EQ_map_2013_es_clear.jpg). (**b**) Guerrero and Oaxaca coast showing rupture areas of the most important earthquakes (1937, 1965, 1978, 1982, 1995, 1996 and 2012) and the estimated location of the 1787 event^8^, used in tsunami simulations (Fig. 4), slip modelling and coseismic deformation models (Supplementary Information and Figs. S9, S10, S11).

**Figure 2:**
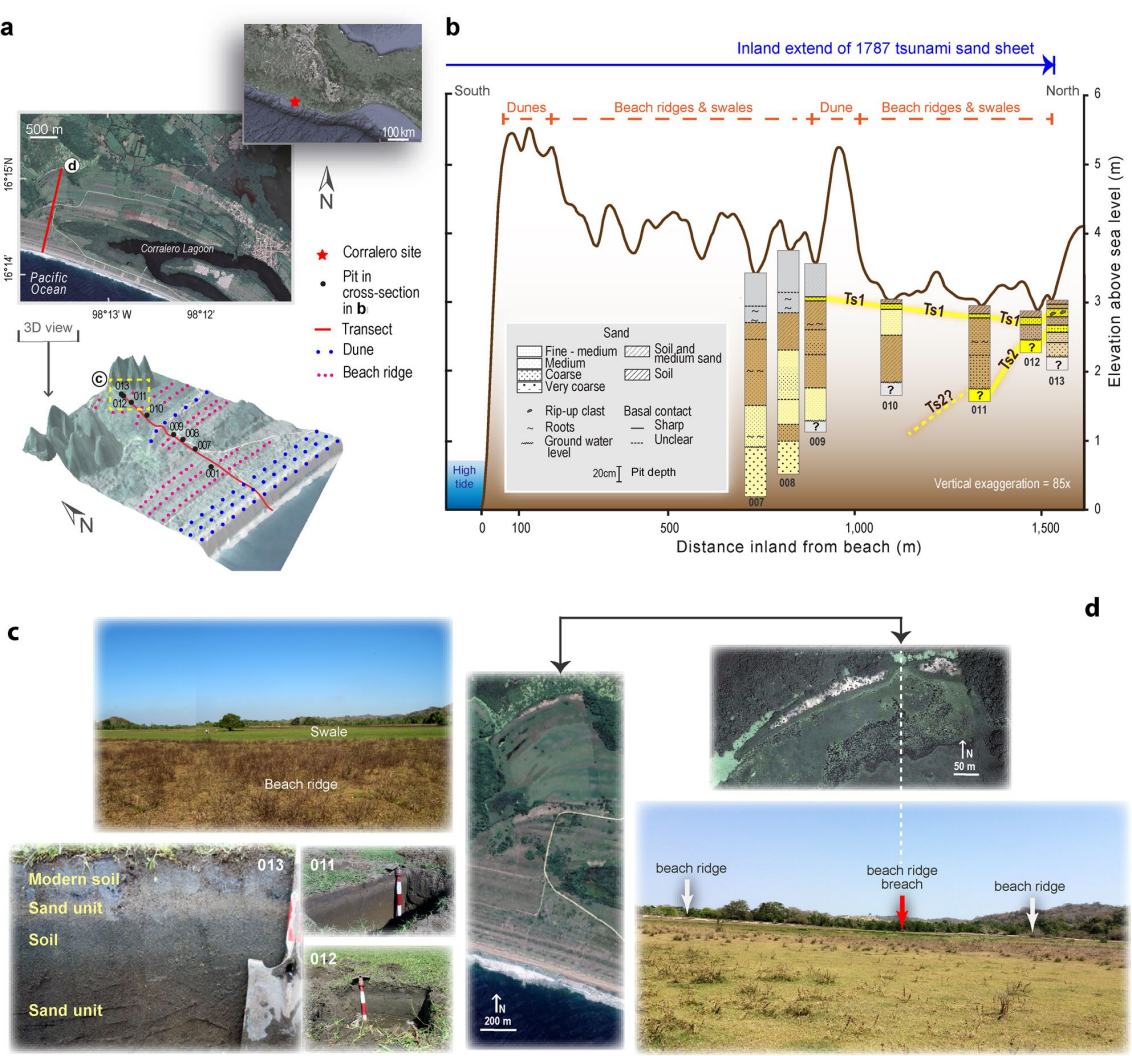
Summary of tsunami deposits and geomorphic evidence of great tsunami flooding in Corralero, Oaxaca. Supporting data in Supplementary Table S1 Supplementary information: Table S2 and Fig. S7. (**a**) Location map of study area and 3-D Lidar-based model (5 m resolution) of the bare surface at Corralero coastal plain, showing sand dunes, beach ridges and swales. (**b**) Cross section of pits, sand units correlated by stratigraphic and sediment characteristics, TSI shows inland extent of 1787 tsunami sand sheet. Microfossil, sediment elemental composition and ^210^Pb dates of pit 013 are shown in detail in Fig. 3. (**c**) Upper picture shows beach ridge and swale (people and a large tree standing for scale); lower pictures show pits at swales with evidence on sand units with abrupt basal contacts and soils beneath and above them. (**d**) Beach ridge breaches shown on satellite images (left and upper pictures) and on field-based photograph (lower picture), indicating tsunami breaching.

Here, we focus on the Corralero coastal plain, in southwestern Mexico, where a great earthquake, M 8.6, triggered a giant tsunami that poured over the coast of Oaxaca, Guerrero, and Chiapas, flooding 500 km along the Mexican Pacific coast and more than 6 km inland, reportedly in 1787^8,12^. Corralero sits on the Oaxaca coast, a segment of the Mexican subduction with frequent earthquakes (every ± 40 years.) and magnitudes ranging from 7.3 to 7.7^13–15^ (Fig. 1a,b). The Corralero coastal plain is a wavy extensive alluvial plain reaching up to 10 km from the coast inland, formed by a series of beach ridges and swales, and a coastal lagoon (Fig. 2a), if indeed a M 8.6 earthquake occurred, a catastrophic tsunami with modelled wave heights of up to 20 m^12^ could overpass a series of 4 to 6 m-height beach ridges (Fig. 2a,b) and flood 6 km inland^8,12,15^ .

We concentrate on the western part of the Corralero plain, west of the Corralero lagoon, where the topography is relatively low (< 6 m high a.m.s.l.) and could have experienced the effects of the 1787 great tsunami, being located approximately half way of the estimated near-trench earthquake rupture^8^ (Fig. 1b). Moreover, the western sector of the Corralero plain has not been affected by local agriculture and other human activities, minimizing the effects of anthropogenic disturbance and uncertainties in the search of tsunami deposits and signs of seismic activity.

## Results

### Historical records of the 1537 and 1787 earthquakes and tsunamis

#### 11 November 1537 earthquake and tsunami

The sixteenth century in Mexico set the fall of the Aztec Empire and of the process of conquest and evangelization by the Spaniards. In this context, the existing documents of the time are of two types: *codices* with iconographic, and chronic representations, mainly written by friars. The fall of Tenochtitlan occurred in the year 1521, in 1524 the first Franciscan friars arrived in the New Spain (Mexico), followed by the Dominicans in 1526 and the first Augustinians in 1533^16^. Although García Acosta and Suárez Reynoso^17^ list a series of pre-Hispanic earthquakes, the first ones that occurred in colonial times, prior to the earthquake in question (1537), are the 1523, 1530, 1532, and 1533 events.

Tsunami reports by the beginning of the sixteenth century in Mexico are even scarcer due to the fact that Spanish settlements in the coastal zone did not become consolidated due, among others, to communication problems and weather conditions. The earthquake of 11 November 1537 can be considered the first for which there is a historic record, although doubtful, and of a tsunami. It is also the first recorded, since the arrival of the Spanish, which mentions damage on the coast of the Mexican Pacific, so it could also be considered the first subduction earthquake reported of the colonial era.

The earthquake of 1537 is mentioned both in *codices* and in some chronicles. Kingsborough^18^ published both the *codices’* images (Supplementary Information 1) and the paleography of his notes: "*This year Six Houses and from 1537, the africans in Mexico City wanted to rise up […] The star was smoking and there was an earthquake, the greatest of which I (Pedro de los Ríos, Dominican monk) have seen, although I have seen many from these parts*.” From the Augustinian chronicle, we now know the exact date on which the earthquake occurred and we have the description of its effects, at least in the region corresponding to the province served by the Chilapa-Tlapa monasteries. Grijalva’s work^16^, written approximately in the year 1628, very much takes the description of the effects of the earthquake from a manuscript written by Fray Agustín de Coruña “*History of the spiritual conquest of those regions*”, and of which he records their existence (p. 509). From Grijalva's description^16^, we know that: the church and the fence of the garden totally collapsed; the tremors were continuous, and there were mudslides and mudflows in the Sierra Madre del Sur, and landslides that were flown by streams and rivers. The report of the tsunami on the coasts of Mexico appears in the *Table of Tsunamis Caused by Earthquakes in the Pacific Region Except near Japan*^19^, pointing that it was produced by an earthquake in Mexico, and it is conclusive when mentioning the damage in the coastal region of Mexico. However, Iida et al.^20^ mistakenly recorded a source in the Chilean tsunamigenic zone (Q). Thus, this is the reason why the tsunami is not Trans-Pacific, but it was generated in the Guerrero-Oaxaca area of Mexico. Finally, Soloviev and Go^21^ located the earthquake epicentre in Mexico, and the tsunami on the coast of Mexico, although they rate it as questionable, that does not exclude that it did occur. Therefore, the mistake made on wrongly locating the tsunami source in Chile made some authors doubt on the reliability of the information. In neither case, the original source of information is recorded.

#### 28 March 1787 earthquake and tsunami

On Wednesday, 28 March 1787, between 11 am and 12 pm local time, the first of a series of earthquakes was felt on the southeast coast of the Mexican Pacific, in central Mexico and even in the state of Veracruz next to the Gulf of Mexico. According to the chronicles of the time^22,23^, the most intense of the earthquakes occurred on the 28th March (M = 8.6)^8^ and on 3rd April (M = 7.3)^24^ during the morning. In both cases, tsunamis were reported and included in national^25,26^ and international catalogs^21,24,27^.

Below, we describe the 1787 event and in Supplementary Information 1 present the exact quotations obtained from original documents, which in many cases have been cited incompletely and subsequently cited by other authors.

The series of earthquakes that occurred between March 28 and April 3, 1787, are called the San Sixto Earthquakes^28^ Earthquakes. However, Núñez-Cornú^15^, Núñez-Cornú et al.^12^ and Suarez and Albini^8^ assign this name to the first earthquake, which occurred on March 28 at 11:30 a.m.

#### 28 March 1787 tsunami

Acapulco behaviour of the sea on 28 March was described by the Rafael Vasco^29^: *by* ~ *12: 00 h the sea began to withdraw and grow, without waves or particular disturbance, at 14:00 h the phenomenon increased, in 4 min it dropped* ~ *3 m and the same rose in 6 min. The beach was uncovered* ~ *90 m repeatedly, and it was increasing; at 4:00 p.m. the sea rose* ~ *4 m, overflowing the pier and flooding houses near the beach; 17:00 h the sea flow decreased, each movement took 15 min. The phenomenon lasted 24 h, until the sea settled back*. From the description of the tsunami flood ^29,30^ and using a 1712 map of Acapulco (Supplementary Information 1), it is possible to know the location of the places mentioned in the text. An 1889 old map of Acapulco with a topographic profile (Supplementary Information 1), it is possible to estimate that the tsunami flood must have been at least 4 m above sea level, and being able to reach the houses and streets closest to the beach and the pier. While, du Petit-Thouars^31^ and Belcher^32^ indicate that the sea retreated to the rocks located in the middle of the bay, Punta Manzanilla, leaving them dry, and that the Philippine Nao, which was anchored ~ 17 m deep, showed it reduced to just 6.68 m (i.e. the sea dropped 10 m). In the report sent from Igualapa (Ometepec jurisdiction) the following events are described, narrated by survivors ^23^: *the sea withdrew more than 4 km, returned again, left thousands of fish in the place that was left without water, and that the sea threw eleven people and left them hanging and stuck in the poles of a mountain, at a distance of* ~ *6 km and at "excessive height"*. According to Gay^28^, “… *the sea withdrew more than 4 km and returned with the speed with which it had moved away and covered with its waves the forests of the beach, reaching a distance of more than 8 km, some fishermen perished and others were saved badly damaged*”.

#### 3 April 1787 Tsunami

The 3 April 1787 earthquake produced greater damage in Tehuantepec, and although it is not completely clear in the report published by the Gazette of Mexico ^23^, “…*it produced an alteration in the sea near Tehuantepec, accompanied by bellows and which throws fish and shells onto the beach*”; Orozco and Berra ^25^ added that this phenomenon also occurred on the coast between Pochutla and Juquila.

### Tsunami deposits

**Figure 3:**
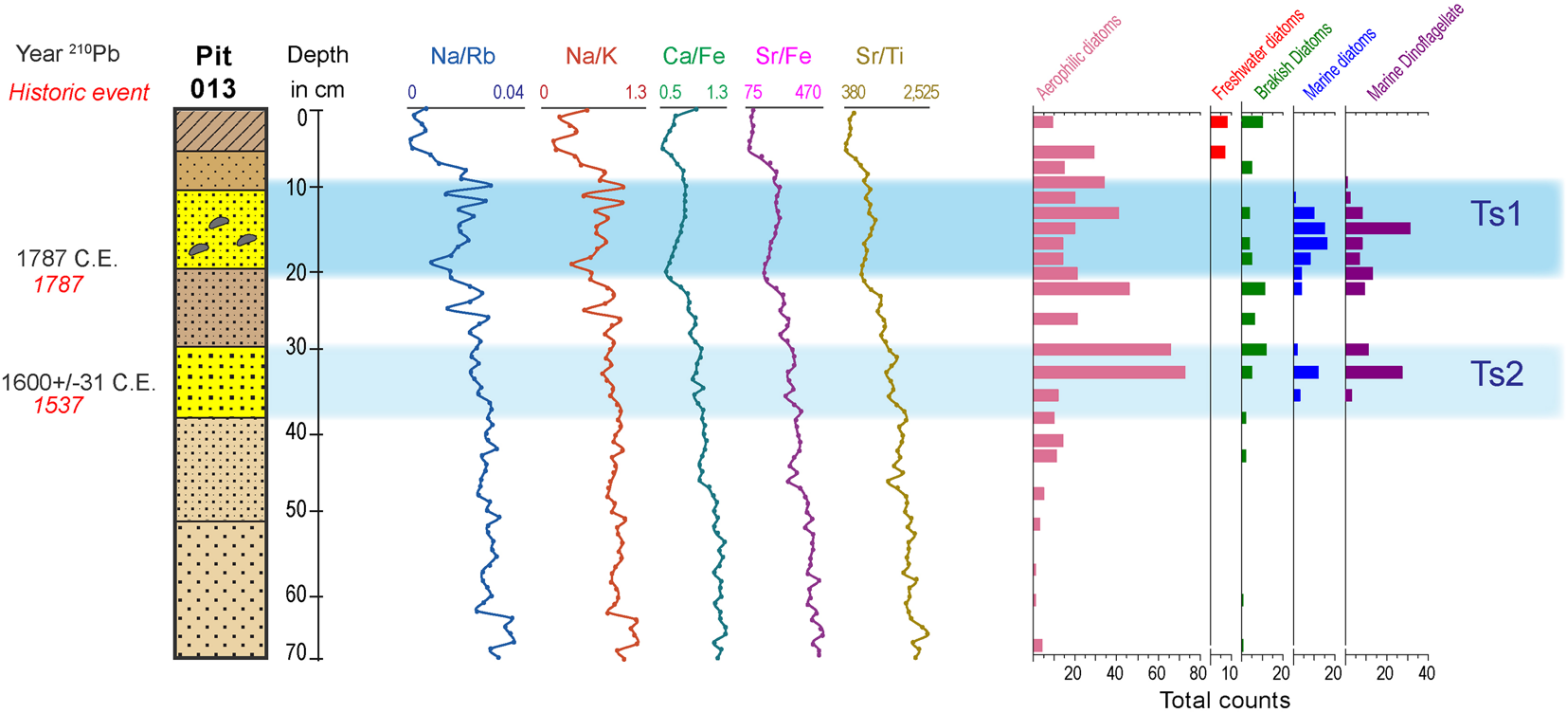
Evidence of great tsunamis at Corralero. Cross-section of pit 013. Yellow sand units indicate tsunami deposits TS1 and TS2 related to the great 1787 earthquake (M8.6)^8^ and tsunami (Supporting data in Supplementary Table S2 and Supplementary Fig. S7) and the probable 1537 earthquake and tsunami, respectively. Elemental composition of sediments shows increase of element ratios indicative of marine flooding (Supplementary Fig. S8). Diatom assemblages and dinoflagellate indicative of marine origin suggest tsunami deposits related to the great 1787 and the 1537 earthquakes (Supporting details in Supplementary Fig. S7, Supplementary Information 1).

We searched for tsunamigenic deposits on the Corralero coastal plain, bounded to the east by a lagoon with a 300 m wide sand bar barrier. These coastal plain features a beach, beach dunes up to 5.5 m high, a series of beach ridges and swales, and marshes around the lagoon (Fig. 2a). The beach ridges on this side of the lagoon extend up to 2 km inland. We studied sediment characteristics from 13 locations on swales between ridges reaching up to 5 m height a.m.s.l. along a transect 1.5 km inland looking for tsunami deposits and signs of seismic activity. The first sign of tsunami deposit is a 5-cm thick lens of medium to coarse sand with an abrupt basal contact with an organic sandy soil below (Fig. 2b,c). From here on this sand layer is continuous up to 1.5 km inland where the 10 cm-thick deposit contains rip-up clasts. We focused on the sediment results (site 013 and 013A, the latest located ~ 200 NNW from 013) furthest inland located 1.5 km away from the shoreline because it provides evidence of the tsunami extent of flooding and we correlate these results with the tsunami deposits found towards the shore (Fig. 2, Supplementary Table S1). Site 013 is located in a swale 3 m a.m.s.l. (Fig. 2b,c) and shows 7 sediment units (Figs. 2b, 3), of which a remarkable light brown (brownish yellow) layer composed of medium to coarse sand containing rip up-clasts (TS1) shows an abrupt basal contact at about 20 cm depth with the unit below it—a dark brown medium sandy to silty incipient soil; and second noteworthy layer (TS2) consisting of medium brown sand at 30 to 41 cm depth with an apparent sharp basal contact with the unit below. Grain size statistics (Supplementary Figure S6) also show two beds where the proportion of sands increases. Site 013-A show two units characterized by a higher sand content (more than 50%). The first, between ~ 10 and 20 cm deep, is characterized by an average size of coarse-medium sands, very poorly classified with a very platykurtic curve. The second unit, between ~ 30 and 40 cm deep, formed by medium sands and with a leptokurtic distribution. The first unit (TS1), at site 013 between ~ 8 and 16 cm, is more evident given that it shows an increase in the average grain size (medium sands), is very poorly classified, and with a very leptokurtic curve; at the base of 013, only the upper part of unit two (TS2), between ~ 26 and 30 cm, is marked by an increase in the average grain size (medium sands), very poorly classified and with an extremely leptokurtic curve. Although kurtosis values tend to be extreme, in both cases they could be related to a highly energetic environment as the one responsible for this condition^33,34^. A line of characteristics suggests tsunamigenic origin of these sandy layers (Fig. 2b). First, both sand units have sharp basal contacts with the units below and unit TS1 contains rip-up clasts implying a sudden and energetic event. Second, both units contain higher abundance of marine diatoms and dinoflagellate which are absent in the adjacent units below and above them (Fig. 3, Supplementary Table S2, Supplementary Fig. S7). Third, the elemental composition of these units shows relatively higher content of Na/Rb, Na/K, Ca/Ti, Ca/Fe, Sr/Fe, Sr/Ti, Ba/Ti, Ba/Rb ratios indicative of marine influence (vs. terrestrial source) (Fig. 3, Supplementary Fig. S8). These characteristics support the allochthonous origin of sand units TS1, more remarkably, and TS2. Besides, the LIDAR-based model, satellite image, and field observations suggest some of the beach ridges show evidence of breaches and scour (Fig. 2d). Hence, considering the abovementioned characteristics, we deduce that in particular sand unit TS1, and possibly TS2, correspond to marine sediments that were transported from the nearshore and into the coastal plain. Only an extremely powerful tsunami would be capable of scour and breaching sand-dunes and beach ridges > 5 m high, transporting sediments from the nearshore and depositing them in the swales as far as 1.5 km inland. These makes the first geologic evidence of great earthquakes and tsunamis of a subduction zone previously underestimated as potential source for great earthquakes and tsunamis.

### Dating

We applied radionuclide ^210^Pb dating, suitable for century-scale dating, on a 50 cm-long sediment core from site 013 to calculate the sedimentation rates and estimate the timing when units TS1 and TS2 were deposited. Assuming a constant sedimentation rate, the ^210^Pb measurements indicate that sediments at 19.59 cm depth have a depositional age of 224 years, i.e. between the 1804 and 1771 C.E., with a more probable deposition in C.E. 1787 year (see Methods section, Fig. 3). This timing agrees with the reported date of the great earthquake and tsunami on 28 March 1787^8,12,15^ (see Supplementary Information 1), consequently suggesting the temporal link between the tsunami event described in historical records (see Supplementary Information 1) and the abovementioned TS1 tsunami deposits.

Potential TS2 tsunami deposits at ~ 36 cm depth, and the layers above and below it, lack organic material for radiocarbon dating, and is beyond the scope of ^210^Pb dating method, upper limit between 110 and 155 years^35^, even though, we calculated the uncertainties of the time range and the age of this sediment unit and estimated 411.7 ± 31 y suggesting deposition between years 1,630 and 1569, i.e. 1,600 ± 31 years, assuming a constant sedimentation rate. Several earthquakes fall in this temporal range (1591, 1604, 1608), though we found no mention of a tsunami in local historical records. However, a strong earthquake is reported in 1537 on the Guerrero coasts and a Japanese catalogue mentions that the Mexican coast was affected by a tsunami on this date^19–21^.

The impact of the 1787 tsunami on the Corralero coastal plain is further supported by the presence of beach ridge breaches near 730 and 1,500 m from the shoreline (Fig. 2d). These two ridges made of sand dunes with loose sediments and heights of 5 and 3 m respectively, have in common the occurrence of scour on the seaward and landward sides (Fig. 2d). Currently the presence of ponds on both sides suggest the landward directed outflow from the tsunami^36–38^. This observation suggests that the 1787 tsunami approached the coast from SSE to NNW (Fig. 2d).

**Figure 4:**
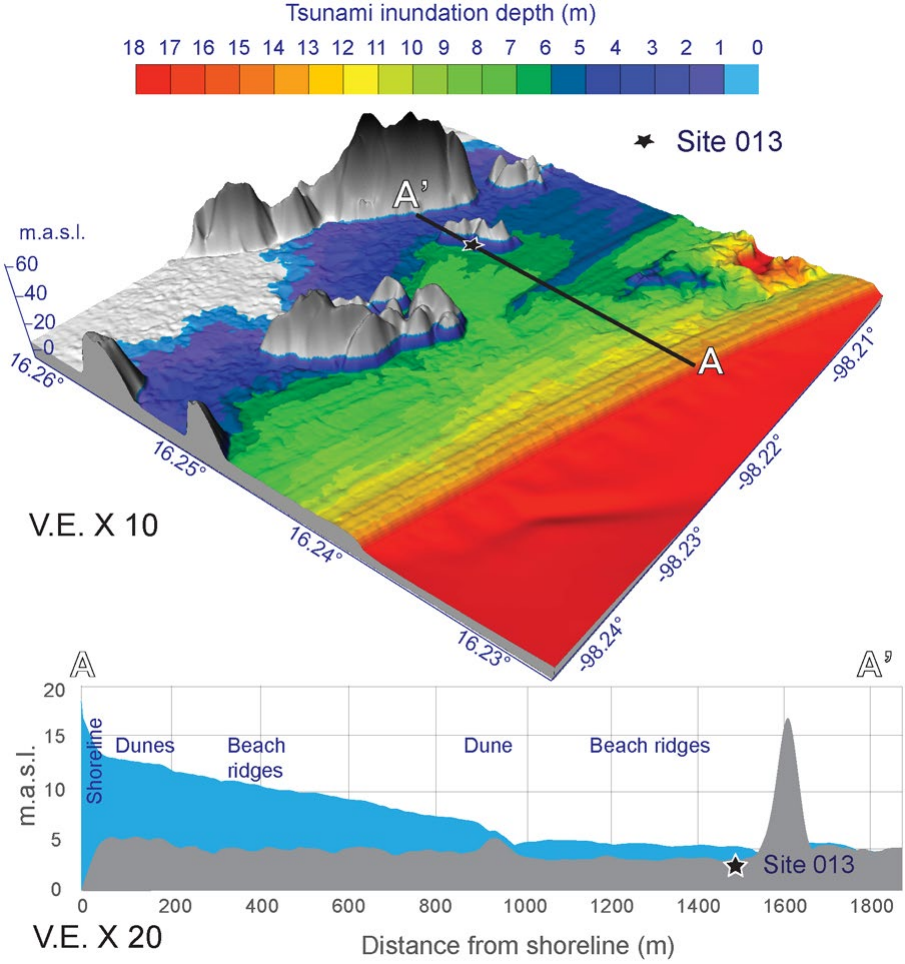
Tsunami inundation depth model. Tsunami inundation depth model based on hypothetical earthquake estimated rupture area^8^ shown in Fig. 1b. (**a**) Tsunami inundation depth showing ~ 5 m depth at site 013. Tsunami inundation in this model can reach up to 5 km distance in flat areas which is accordant with historical documents (Supplementary Information 1). (**b**) Tsunami inundation depth and bare terrain profile.

Thus, a set of key observations point out to an SSE-approaching tsunami and caused a catastrophic inundation of the beach ridges coastal plain oriented WNW-ESE, almost perpendicular to the tsunami flow direction. The position of beach ridges breaches and respective scour^38^ reflect a preferential energetic flow from the SSE from the tsunami that spilled over the coastal plain. This is also compatible with a tsunami model, developed to test tsunami inundation depths, showing tsunami directivity to the NNW and higher tsunami waves than 18 m at Corralero plain^12^ (Fig. 4).

Based on the maximum distance from the current shoreline inland (1.5 km) at which we traced the 1787 tsunami deposits, and the current elevation of sand dunes and beach ridges (up to 5 m above sea level), plus tsunami inundation depth based on modelling (Fig. 4), we estimate a wave runup of more than 5 m at the 013 site, assuming negligible relative sea-level uncertainties in the last 200 years because these are likely less than 0.1% of the elevation of current sand dunes and beach ridges overpassed by the tsunami inundation (Fig. 4).

### Stochastic heterogenous slip models and vertical coseismic deformation of the 1787 earthquake

Whether the landscape is uplifted or subsides is dependent on the location of slip on the megathrust. Broadly speaking, the area overlying a slip patch will move up, whereas subsidence will occur primarily downdip of the slipping region. In fact, due to the short continental shelf, in Mexico it is not uncommon for the coastline to be above the part of the megathrust that slips coseismically (e.g.^39^) and thus has uplift, not subsidence. So, it’s important to understand whether, given the expected rupture area of the 1787 event, subsidence is likely. Based on the diatoms above and below tsunami deposits, we attempted to infer land-level change. (Fig. 3, and Supplementary Fig. S7). However, in humid tropical climate diatoms are relatively scarce if compared with environments at mid-latitudes, where most of the studies of land-level changes using diatoms come from. Even so, we observe slightly more brackish diatoms and no freshwater diatoms above both TS1 and TS2 (Supplementary Fig. S7). However, we cannot preclude with absolute certainty the nature of a land level change concomitant with the 1787 event, nor for the 1537 event, from microfossils. To address this we generated 600 stochastic slip models^40^ in a broad magnitude range within the expected rupture region and calculated the expected coseismic vertical deformation (Supplementary Figs. S9 and S10). The purpose of the stochastic models is not to constrain the size of the earthquake. Rather their purpose is to explore, given that what is known about the event has large uncertainties, what is the relative likelihood of uplift or subsidence at the study site given different source magnitudes and dimensions. The only thing we assume for these models is (i) the down-dip limit of slip which is fairly well known from observations of modern large events and (ii) that the sources be loosely centred around the source region proposed by Suarez & Albini^8^. Then the 600 stochastic models are by design built to sample a range of possible magnitude values (7.8 to 9.1), and lengths (from 150 to more than 500 km). These lengths are not arbitrary they are obtained from known source-scaling relationships^41^. For a given magnitude there is an empirical probability density function that specifies the likely lengths, and we use this sampling approach to define the length for any given source. We find that the resulting vertical deformation depends strongly on the unknown details of the heterogenous slip distribution, however, in the magnitude range M8.5-M8.7 65% of the models lead to co-seismic subsidence (Supplementary Figs. S10 and S11). So, after all this, what the results show is that it is not unreasonable to expect subsidence at the site even if the magnitude of the event is not exactly 8.6 or if the source dimensions are not exactly what was inferred by Suarez and Albini^8^.

## Discussion and conclusions

Tsunami deposits from the last 500 years in the Corralero- Oaxaca coast thus imply large (> Mw 8.6) earthquakes and tsunamis than previously considered in tsunami hazard assessments for the Mexican Pacific coast parallel to the subduction zone. Until now instrumental data report interplate earthquakes that produce relatively small tsunamis by medium size earthquake ruptures near the coast, thus tsunami hazard has been underestimated^42^. However, the short-time span of instrumental and historical records and perhaps the long recurrence of large earthquakes has limited the tsunami and earthquake^43,44^ hazard assessment in this and other subduction zones of the world. Our observations and historical observations, however, require a tsunamigenic earthquake, resulting from a long-fault rupture near the trench on the shallow portion of the interface (see Supplementary Information and Fig. S9), such as in Sumatran megathrust earthquakes of 1797 and 1833^45^, the 2010 Maule earthquake^46,47^, and the 2011 Tohoku-Oki earthquake^48^.

A long-fault rupture ca. 450 km long in 1787 broke again as small ruptures and medium size earthquakes (Mw 7.3 to 7.7)^13–15^ in 1937, 1965, 1968, 1978, 1995 and 1996, and 2012^8,13–15,49^. Our observations based on a combination of geologic and historic evidence, together with modelling results, imply that this subduction zone is also subject to long-fault ruptures near the trench capable of producing large tsunamis (Fig. 4). Because microfossil evidence is scarce to infer land-level changes, stochastic modelling of the 1787 spatial slip distribution revealed the spatial complexity of the 1787 earthquake slip and the spatial slip distribution, and helped to estimate the vertical deformation (See Supplementary Figs. S9, S10, S11). Although the exact details of the 1787 event remain unknown, it is still important to explore if, given what is known about it, and about the Mexican subduction zone, our interpretation of the geological observations is seismologically consistent. The coseismic modelling shows that even if the details of the event are uncertain coseismic subsidence is not unlikely. The tsunami modelling further shows that under reasonable conditions it is possible to inundate to the observation site. We do not claim that either of these modelling efforts constrain the exact details of the earthquake, but simply that they show parsimony with other known seismological characteristics and facts.

We showed here that a massive tsunami, with tsunami waves as high as 18 m, affected the coastline of Oaxaca in 1787 that flooded the Corralero portion of this coast at least as far as 5 km inland, most probably causing coseismic coastal subsidence, and we have determined its most probable source: a long-fault rupture tsunamigenic earthquake. Our observations also indicate that indeed subduction zones might have variable rupture modes, long- and short-fault ruptures, along the Middle American trench. This new geologic evidence shows that this zone is capable of generating great tsunamis with devastating effects. We infer that the recurrence interval of such great earthquakes and tsunamis, though historical records of the 1537 event are scarce, the 1537 tsunami was likely a predecessor, judging by the location of the tsunami deposit more than 1.5 km inland, and propose a ca. 250-year recurrence. Our data validate incomplete historical records of two great tsunamigenic earthquakes and suggest that the Oaxaca-Guerrero part of the Mexican subduction zone is capable of producing long-fault rupture by great earthquakes, and tsunamis. Geologic evidence aid in a better understanding of the occurrence and recurrence of near-trench, long-fault rupture earthquakes and great tsunamis. It allows to realistically assess the hazard by great earthquakes and tsunamis, and near- and far-field effects along this and other subduction zones less frequently affected by these events.

## Methods

### Field survey

This study initiated with a short-5 day-field reconnaissance following historical descriptions of the 1787 earthquake and tsunami^17^. We explored for suitable sites with potential for tsunami deposits preservation near the mangrove marshes, at small ponds near the coast, and at salt pans around the eastern side of Corralero lagoon (formerly Alotengo lagoon). We found these sites were disturbed by human activities and no sand beds interbed with soils. Building on previous reconnaissance, we later searched for 1787 sand sheets along 2 transects at Corralero coastal plain by digging test pits, using a hang auger and piston corer into ridges and swales at 23 sites (Fig. 2a, Supplementary Table S1). We found evidence of tsunami sands at sites along the two parallel transects dug into swales between beach ridges as far as 1.5 km inland (Fig. 2a,b).

We state that no humans were involved during field survey, or any questionnaire has been conducted for information regarding the study.

### Stratigraphic-sediment analysis

We assembled stratigraphic cross-sections from correlated pits and piston corer, estimated visually particle size and described the basal contacts between stratigraphic units (Fig. 2b). We subsampled a monolith 70 cm long recovered from pit 013A, located 1.5 km inland (Fig. 2a,b) and recovered a parallel piston corer 013a, 65 cm long, additionally, a 30 cm core was collected from a short distance, 200 m, from site 013. Monolith 013A was subsampled for detailed microfossil (diatoms, dinoflagellates and foraminifera), geochemical analysis, and 210Pb dating. Core 013a was used for magnetic susceptibility measurements.

### Grain size

We collected a complete slice, using a square gutter to preserve the whole monolith, from the wall of pit (013A) and a core (013) from another pit located 200 m apart (013A is to the NNW from 013). Then at the lab we subsampled at 2 cm intervals 37 samples from slice 013A and 16 samples from core 013, with a 2 cm interval. Samples were processed in a Mastersizer 2000 particle laser diffractor. Statistics were calculated using the GRADISTAT^50^ program applying Logarithmic graphical measures^51^.

### Diatoms, dinoflagellates and foraminifera analysis

Samples for microfossil (diatoms and dinoflagellates) analyses were collected from 013 core at discretionary depths (Fig. 3, Supplementary Table S2, Supplementary Fig. S7). Dry sediment (1 g) was sampled from each horizon and dispersed in still water. Thin sections were made with 400 μl of liquid dispersion for each slide. Counts of valves were undertaken with a Carl Zeiss (series 470,801–9,097) microscope, and species composition is reported as total abundance recognized in the slides. Microfossil diagrams were designed using Tilia software.

Samples for foraminifera analysis were collected from core 013 at 1 cm interval. Sediments for benthic foraminifera were washed with current water through a 63-micron sieve, to separate them from the matrix and eliminate silts and clays. The washed residues were air—dried, weighed and analysed through a binocular microscope to obtain the BF. Benthic foraminifera were absent in all analysed samples.

### Element concentration analysis and ^210^Pb chronology

For element concentration analysis, dry and ground sediment samples were placed in low-density polyethylene cells (bottom covered with Prolene® film), compressed manually by using a Teflon® rod, and analysed by X-ray fluorescence spectrometry (XRF, Spectrolab Xepos-3) under He atmosphere.

In order to estimate the sediment accumulation rates (SAR) and the age model of the core, ^210^Pb was determined through its radioactive descendant ^210^Pb by alpha spectrometry (Ortec Ametek Ensemble)^52^. The ^210^Pb-derived chronologies were calculated using the constant flux (CFCS) model^53–55^, and uncertainties were estimated. Analytical quality control included the evaluation of analytical blanks, the assessment of precision by replicate analysis (n = 6) and the assessment of accuracy through the analysis of reference material: IAEA-158, IAEA-405, and IAEA- 433 for XRF, and IAEA-300 for ^210^Pb and ^137^Cs.

### Stochastic heterogenous slip models and vertical coseismic deformation

We produced 600 stochastic heterogenous slip models in the magnitude range M8.0 to M8.8 using the method described by Melgar et al.^40^. We used a three-dimensional slab geometry of the megathrust^56^ and assumed that the down-dip edge of slip occurs at 35 km depth. This assumption is consistent with the down-dip edge of slip imaged in slip inversions of previous large earthquakes in Mexico^39,57,58^. For each slip realization we fixed the centre of the fault to the area proposed by Suarez and Albini^8^ but allowed the length and width to vary according to source dimension scaling laws^40^. The pattern of slip is from a Von Karman correlation function with correlation lengths following scaling laws that depend on the length and width the causative fault^59^. For each heterogenous slip distribution we assumed an elastic half space with rigidity of 30GPa and calculated the vertical deformation using known analytical solutions^60^.

### Tsunami inundation depth model

We use tsunami inundation depth modelling to determine potential tsunami spatial inundation pattern based on the current topography. Other components that influence the complex tsunami inundation process were not included, such as tsunami directivity, flow velocity, pre-tsunami topography, surface friction produced by vegetation coverage, among others. We did not aim at the determining the earthquake source because of the sparse historical records and details of the earthquake occurrence, which are not enough to determine the exact earthquake source from tsunami models^61^. Nevertheless, this model aimed to confirm potential areas where a tsunami flooded and deposited a sand layer.

The inundation depth model was calculated using Cost-Distance Algorithm (CDA) and an empirical interpolation model. The CDA calculates the least accumulative cost distance for each cell to the nearest source over a cost surface, in this case the terrain model. The shoreline was assumed as the source, and a parallel line was drawn at 10 km (destination) from the shoreline to force the model to run in a perpendicular direction from the coast—inland. The CDA shows the cost of travel to any point inland, resulting in a continuous raster model with values of Cost Distance (CD).

The topographic input consisted of a LIDAR DTM, 5 × 5 m resolution. To calculate the tsunami inundation depth (TID), an empirical linear interpolation was applied to convert the values of CD into a value of inundation depth (meters above the ground). It was assumed that at the shoreline the maximum tsunami amplitude (MTWA) reached 18 m^12^ and at site 013, i.e. horizontal inundation reference point, the probable inundation depth reached ca. 5 m.

The tsunami inundation modelling was conducted only by the assumption of 18 m of tsunami amplitude on the shoreline reported by historical documents and cited by Núñez-Cornu et al.^12^. Therefore, tsunami aspects such as directivity, flow direction, and flows speed were not considered. The model represents the required tsunami inundation depths to produce a 10 cm tsunami deposit on the site 013, considering that this is the minimum deposit thickness since climate factors (e.g. rain, wind) might have eroded it since deposition. Specifically, the assumption to assign the inundation depth value of 5 m on site 013 was based following Goto et al.^62^ observations in Sendai plain where they found that the sediment concentration in the tsunami inundation flow can be approximated at 2% of the inundation depths.

## Supplementary information

Supplementary file1 (PDF 4378 kb)
