# Supplementary material for: Sand deposits reveal great earthquakes and tsunamis at Mexican Pacific Coast
Source: Sci Rep. 2020 Jul 10;10:11452. doi: 10.1038/s41598-020-68237-2 (PMC7351727; doi:10.1038/s41598-020-68237-2)
Supplement: Supplementary file 1 — Supplementary file1 (PDF 4378 kb) [file 41598_2020_68237_MOESM1_ESM.pdf]

**Sand deposits reveal great earthquakes and tsunamis at Mexican Pacific Coast**

María-Teresa Ramírez-Herrera[1*], Néstor Corona[2], Jan Cerny[1], Rocío Castillo-Aja[3], Diego Melgar[4], Marcelo Lagos[5], Avto Goguitchaichvili[6], María Luisa Machain[7], Miriam L. Vazquez-Caamal[8] , María Ortuño[9], Margarita Caballero[10], Ericka Alinne Solano-Hernandez[8], Ana-Carolina Ruiz-Fernández[7]

1. Laboratorio de Tsunamis y Paleosismología, Instituto de Geografía, Universidad Nacional Autónoma de México, México. tramirez@igg.unam.mx
2. COLMICH, Centro de estudios de Geografía Humana, Michoacán, México; and Laboratorio de Tsunamis y Paleosismología, UNAM.
3. Depto. de Geografía y Ord. Territorial, CUCSH. Universidad de Guadalajara, México
4. Department of Earth Sciences, University of Oregon, Oregon, USA.
5. Instituto de Geografía, Laboratorio de Tsunamis, Pontificia Universidad Católica de Chile, Santiago, Chile
6. Instituto de Geofísica, Universidad Nacional Autónoma de México, Unidad Michoacán, México
7. Instituto de Ciencias del Mar y Limnología, Universidad Nacional Autónoma de México, México
8. Universidad del Mar, Ciudad Universitaria, Puerto Ángel, Distrito de San Pedro Pochutla, Oax., México
9. Ciencias de la Tierra, Universidad de Barcelona, España
10. Instituto de Geofísica, Universidad Nacional Autónoma de México, México

**Supplementary Table S1. Surveyed sites.**

We began finding evidence for 1787 tsunami and pre-1987 tsunami at Corralero coast plain on the second field survey; these sites are marked in the table below in green. We searched previously, i.e. during the first field survey, unsuccessfully in mangroves, salt-pans and on beach-ridge plains where swales have been altered by agriculture and farming, and near the lagoon by salt extraction activities. These sites are marked in yellow.

| Survey/ site name | Coordinates | | Observations |
|---|---|---|---|
| **1st field survey** | | | |
| Alotengo East | 16°12'20.53"N | 98° 8'12.32"O | Evidence obliterated |
| Alotengo swale | 16°12'7.07"N | 98° 8'45.53"O | Evidence obliterated |
| Al-01-07 pond | 16°12'14.99"N | 98° 9'41.25"O | 210Pb sedimentation rate at 25 cm: 0.258 cm/year |
| | | | |
| **2nd field survey** | | | |
| COR 01 | 16°14'30.98"N | 98°13'38.28"O | Evidence obliterated |
| COR 007 | 16°14'37.57"N | 98°13'35.51"O | Evidence obliterated |
| COR 008 | 16°14'40.74"N | 98°13'34.57"O | Evidence obliterated |
| COR 009 | 16°14'42.40"N | 98°13'34.36"O | Sand sheet |
| COR 010 | 16°14'48.84"N | 98°13'32.16"O | Sand sheet |
| COR 011 | 16°14'54.60"N | 98°13'29.14"O | Sand sheet |
| COR 012 | 16°15'1.33"N | 98°13'27.34"O | Sand sheet |
| COR 013A | 16°15'2.09"N | 98°13'24.89"O | Sand sheet |
| COR 013 | 16°14'55.45"N | 98°13'23.91"O | Sand sheet |
| | | | |
| **3rd field survey** | | | |
| T1 | 16° 14.283'N | 98° 13.368'W | Evidence obliterated |
| T3 | 16° 14.427'N | 98° 13.321'W | Evidence obliterated |
| T4 | 16° 14.587'N | 98° 13.269'W | Evidence obliterated |
| T4a | 16° 14.616'N | 98° 13.258'W | Evidence obliterated |
| T4b | 16° 14.654'N | 98° 13.247'W | Sand sheet |
| T6 | 16° 14.773'N | 98° 13.207'W | Sand sheet |
| T7 | 16° 14.824'N | 98° 13.185'W | Sand sheet |
| T8 | 16° 14.884'N | 98° 13.170'W | Sand sheet |
| T9 | 16° 14.969'N | 98° 13.141'W | Sand sheet |
| T10 | 16° 15.007'N | 98° 13.129'W | Sand sheet |
| T11 | 16° 15.078'N | 98° 13.106'W | Limited access |

**Supplementary Information 1**

**Original Historical Accounts of the 1537 and 1787 March-April Earthquakes and Tsunami**

This supplement presents graphic and written descriptions of the 1537 and 1787 earthquakes and tsunamis. Some of the accounts were summarized by other authors and recited again, though we present here the original sources. The originals were written in Spanish which we reproduce beside a literal translation into English (prepared by Ramirez-Herrera and Castillo-Aja).

**1537 earthquake and tsunami**

The 1537 earthquake is mentioned both in codex and in some chronicles. The textual quotes in this regard are listed below:

***Record 1***

Codex Telleriano-Remensis

Supplemental Figure S1. Codex Telleriano-Remensis. Please see figure at http://www.famsi.org/spanish/research/loubat/Telleriano-Remensis/page_45r.jpg, last access: 15 March 2020

Source: Loubat, J. F. Facsimilies of the Codex Telleriano-Remensis. (2006). http://www.famsi.org/research/loubat/Telleriano-Remensis/thumbs0.html, last access: 31/Mar/2020

***Record 2***

Kingsborough (1831) included in his book both, the *codex* Telleriano-Remensis images and the palaeography, in his notes:

"*This year Six Houses and from 1537, the Africans in Mexico City wanted to rise up [...] The star was smoking and there was an earthquake, the greatest of which I (Pedro de los Ríos, Dominican monk) have seen, although I have seen many from these parts*. "

*En este año de 6 Casas y de 1537 se quisieron alzar los negros en la Ciudad de México de los cuales, áhorcaron a los inventores de ello. Humeava la estrella y uvo un temblor de Tierra el major que yo he visto, aunque he visto muchos por estas partes.*

Source: Kingsborough, Lord. (1831). *Antiquities of Mexico: Comprising fac-similes of ancient Mexican paintings and hieroglyphics, preserved in the Royal Libraries of Paris, Berlin, and Dresden; in the Imperial Library of Vienna; in the Vatican Library; in the Borgian Museum at Rome; in the Library of the Institute at Bologna; and in the Bodleian Library at Oxford. Together with the Monuments of New Spain, by M. Dupaix: With their respective scales of measurement and accompanying descriptions. The whole illustrated by many valuable inedited manuscripts*: Vol. V. p. 155. Published by Robert Havell 77, Oxford Street.

***Record 3***

Orozco y Berra (1888) cited Kingsborough (1831), and from Orozco y Berra´s (1888) work also Milne, 1912, Soloviev and Go (1975), García-Acosta and Suárez Reynoso (1996), and Sánchez Devora and Farreras (1993) re-cited his work.

***Record 4***

García Acosta and Suárez (1996):

Tierra Caliente, Costa Grande, Gro; Colima.

Catastrophe in Tierra Caliente. Another strong earthquake. Intense in today's Guerrero, especially in Tierra Caliente and Costa Grande. (Also reported in Colima)

Paucic Archive, Earthquakes and tremors in Guerrero.

> *Tierra Caliente, Costa Grande, Gro; Colima.*
>
> *"Catastrófico en tierra caliente. Otro fuerte sismo. Intenso en el actual Guerrero, especialmente en Tierra Caliente y Costa Grande. (Reportado también en Colima)*
>
> *Archivo Paucic, Terremotos y temblores en Guerrero*

***Record 5***

Soloviev y Go, (1975) included in his catalogue:
1537. “There was an earthquake in the Valley of Mexico. It is possible that the source of the earthquake was in some adjacent state: Guerrero, Puebla or Veracruz (Milne, 1912 b: Montandon, 1962). According to a Japanese catalogue (Anon., 1961 b), the coast of Mexico was hit by a tsunami. Iida et al., (1967), quite justifiably, express doubt as to the reliability of these data”.
1537. Mexico. Degree of authenticity Q: questionable (“The description gives reason to believe that an occurrence other than a tsunami took place, but that the possibility of a tsunami is not completely excluded”).

***Record 6***

Iida et al. (1967) reported as a tsunamigenic area, where the tsunami was generated - Chile (Q), and - Mexico as the [tsunami] observation site. In the Effects and Remarks section mentioned: “No details known. Report is considered quite questionable because it is carried by no authorities outside Japan”. They record as sources: Anon, 1961 (JMA) and Kawasumi, 1963

***Record 7***

The Japan Meteorologican Agency (1963) reported: “1537. Epicentre region, Mexico. Remarks: Tsunami attacked the coastal region of Mexico.”

***Record 8***

Ruiz Zavala (1984) described:

"Chilapa de Alvarez, in the central part of the current State of Guerrero, 57 kilometres from Chilpancingo… when, on October 5, Jerónimo de S. Esteban and Agustín de Coruña arrived in the area. Church and convent were destroyed by an earthquake on November 11, 1537; Rebuilt, a new earthquake damaged the church and destroyed the convent in 1573 (AA II 130/31; GR 49, 86; BS 198). "

AA = Alphabetum Agustinianum. Thomas de Herrera. Typis Gregorii Rodríguez. Matriti, MDCXLIII

GR = Chronicle of the Order of N.P.S. Augustine in the Provinces of New Spain. Juan de Grijalva. Imp. Victoria, S.A. Mexico. MCMXLII

> *"Chilapa de Alvarez, en la parte central del actual Estado de Guerrero, a 57 kilómetros de Chilpancingo… cuando, el 5 de octubre, llegaron a la zona Jerónimo de S. Esteban y Agustín de Coruña. Iglesia y convento fueron destruidos por un terremoto el 11 de noviembre de 1537; reconstruidos, un nuevo sismo dañó la iglesia y destruyó el convento en 1573 (AA II 130/31; GR 49, 86; BS 198)."*

***Record 9***

Grijalva (1985) provided more details:

"There were great opponents for the company in Chilapa and Tlapa, because it seemed to them the most honest, and where Our Lord had to serve the most ... He therefore appointed Father Fray Juan de S. Román, and Father Fray Agustín de Coruña, so that the PS Román stayed in Occuituco, and Father Coruña passed to the provinces of Tlapa... They arrived at Occuituco, and Father San Román stayed there, then Father S. Esteban passed, and Father Coruña with the speed that the *saeta* fired from a brave arm, and it was, and well-tempered bow. They arrived in Chilapa on October 5, the year of 33… "

> *"Hubo grandes opositores para la empresa de Chilapa y Tlapa, porque les parecía la más honrosa, y donde más se había de servir Nuestro Señor…Nombró pues para esto al Padre fray Juan de S. Román, y al Padre fray Agustín de Coruña, para que el P.S. Román se quedase en Occuituco, y el Padre Coruña pasase a las provincias de Tlapa… Llegaron a Occuituco, y quedándose allí el Padre San Román, pasaron luego el Padre S. Esteban, y el Padre Coruña con la velocidad que va la saeta despedida de un valiente brazo, y fuere, y bien templado arco. Llegaron a Chilapa el cinco de octubre, año de 33…"*

"Arriving then at Chilapa they began to visit their converts, and to preach to the Gentiles with such great fervour, that in a short time the Gospel of all those great provinces was seized on one hand and on the other hand: because from Chilapa they went up to Tlapa with all their region and they went down to the southern sea,… In the province of Chilapa and Tlapa there are today six monasteries of my Order, where twenty friars ordinarily reside, in Thlaucozautitlan; there are two benefits in Guamustitlan, another in Olinalá, another in Tiztlan, another in Tonalá, the convent of our Padre Santo Domingo in Tzilacayoapan, one benefit in Ayutla, another benefit in Cacahuamilpa, and another in Tlapegualapan another, in Acatlán another.

> *"Llegando pues a Chilapa empezaron a visitar a sus convertidos, y a predicar a los gentiles con tan gran fervor, que en breve tiempo se apoderó el Evangelio de todas aquellas grandes provincias por una y por otra parte: porque de Chilapa subieron a*

*Tlapa con toda su comarca y bajaron hasta la mar del sur, ...En la sola provincia de Chilapa y Tlapa hay hoy seis monasterios de mi Orden, donde de ordinario residen veinte frailes, en Thlaucozautitlan; hay dos beneficios en Guamustitlan, otro en Olinalá, otro en Tiztlan, otro en Tonalá, el convento de nuestro Padre Santo Domingo en Tzilacayoapan, un beneficio en Ayutla, otro beneficio en Cacahuamilpa, y en el puerto de Atlapulco otro, en Tlapegualapan otro, en Acatlán otro.*

"What he admires the most is that within one year on November 11, 1537, the convent and church fell from a large earthquake, without being nearby in the garden, or in the courtyard, any stone on stone. Many rocks collapsed: the fountains and rivers of all that region seemed to have been turned upside down: because they were made of thick mud: the great river of Tlalcozuauhtitlán, which is very abundant, ran like mud: the alligators all came ashore loaded of mud, because in reality the land actually rose above the water. "

*"Lo que más admira es, que dentro del año puntualmente a 11 de noviembre de 1537, se cayó el convento e iglesia de un grande terremoto, sin que quedase cerca en la huerta, ni en el patio, ni piedra sobre piedra. Muchas peñas se derrumbaron: las fuentes y los ríos de toda aquella comarca parecía que se habían transtornado: porque estaban hechas un barro espeso: el río grande de Tlalcozuauhtitlán, que es caudalosísimo, corría hecho un cieno: los caimanes salieron a la orilla todos cargados de barro, porque en realidad de verdad la tierra subía sobre el agua".*

"The tremors in Chilapa are continuous, as we experience every day with general pity: and these tremors are so great that the most beautiful building that existed in the entire kingdom knocked us down over the years. The demon could have observed this tremor, because it ordinarily happens at such a time; on the occasion of low water and high winds and extremely dry. He would also know that this building was false, because it grew in a hurry and without a teacher. How much would you guess your downfall? This a bricklayer does. "

*"Los temblores de Chilapa son continuos, como lo experimentamos cada día con general lástima: y tan grandes estos temblores, que nos derribó los años pasados el*

*más hermoso edificio que había en todo el reino.  Pudo tener el demonio observación de este temblor, porque sucede ordinariamente a tal tiempo; con ocasión de pocas aguas y muchos vientos y de extremada sequedad.  Conocería también que aquel edificio iba falso, porque crecía con mucha prisa y sin maestro. ¿Qué mucho que adivinase su ruina? Esto un albañil lo hace.”*

**References**


1. Loubat, J. F. Facsimilies of the Codex Telleriano-Remensis. (2006). http://www.famsi.org/research/loubat/Telleriano-Remensis/thumbs0.html, last access: 31/Mar/2020

2. García Acosta, V., & Suárez Reynoso, G. (1996). *Los Sismos en la Historia de México. Universidad Nacional Autónoma de México*, Centro de Investigaciones y Estudios Superiores en Antropología Social y Fondo de Cultura Económica, p. 64.

3. Grijalva, J. de. (1985). *Crónica de la Orden de N.P.S. Agustín en las provincias de la Nueva España: En cuatro edades desde el año de 1533 hasta el de 1592* (1a ed. en la Biblioteca Porrúa). Editorial Porrúa, p. 39, 52, 63-64, 65

4. Iida, K., Cox, D. C., Pararas-Carayannis, G. (1967) *Preliminary catalog of tsunamis in the Pacific Ocean*. Data Report No. 5 HIG-67-I, Prepared for The State of Hawaii and Office of Naval Research under Contract No. Nonr-3748(03). 261 p.

5. Japan Meterorological Agency (1963) Chapter VI. Table of Tsunamis Caused by Earthquakes in the Pacific Region Except near Japan. In: *The report of the Chilean earthquake, 1960.* Technical report of the Japan Meteorological Agency No. 26, March 1963, p. 51. Published by Japan Meteorological Agency, Tokyo. Available at: http://tsunami-dl.jp/document/067#section-fb6f53ee7e879f7f71ee8ba695c9f7fc , accessed: 20 March 2020

6. Kingsborough, Lord. (1831). *Antiquities of Mexico: Comprising fac-similes of ancient Mexican paintings and hieroglyphics, preserved in the Royal Libraries of Paris, Berlin, and Dresden; in the Imperial Library of Vienna; in the Vatican Library; in the Borgian Museum at Rome; in the Library of the Institute at Bologna; and in the Bodleian Library at Oxford. Together with the Monuments of New Spain, by M. Dupaix: With their respective scales of measurement and accompanying descriptions. The whole illustrated*

*by many valuable inedited manuscripts*: Vol. V. Published by Robert Havell 77, Oxford Street.

7. Milne, J. (1912*). A catalogue of destructive earthquakes, A.D. 7 to A.D. 1899*. British Association for Advancement of Science. Seismological Committee.

8. Montandon, F. (1962). Les megaséismes en Amérique. *Revue pour l'étude des calamités*, 38.

9. Orozco y Berra, J. (1888). Efemérides Sísmicas Mexicanas. *Memorias de la Sociedad Científica "Antonio Alzate"*, I (8), 303–541.

10. Ruiz Zavala, A. (1984). *Historia de la Provincia Agustiniana de Santísimo Nombre de Jesús de México: Vol. II.* , p. 308. Porrúa.

11. Sánchez Devora, A. J., & Farreras Sanz, S. (1993). *Catalog of tsunamis on the western coast of Mexico.* Report SE-50. World Data Center A for Solid Earth Geophysics, NOAA, National Geophysical Data Center, Boulder, Colorado.

12. Soloviev, S. L., & Go, C. N. (1975). *A catalogue of tsunamis on the eastern shore of the Pacific Ocean* (1513-1968). Nauka Publishing House. Can. Transi. Fish. Aquat., 5078, 1984, p. 173 and 251.

**1787 March-April Earthquakes and Tsunami**

On Wednesday, 28 March, between 11 am and pm 12 local time, the first of a series of earthquakes was felt on the southeast coast of the Mexican Pacific, in central Mexico and even in the state of Veracruz next to the Gulf of Mexico. According to the chronicles of the time[1,2], the most intense of the earthquakes occurred on the 28 March (M = 8.6)[3] and on April 3 (M = 7.3)[4] during the morning. In both cases, tsunamis were reported and included in national[5,6] and international catalogs[4,7,8].

Below we describe the exact quotations obtained from original documents, which in many cases have been cited incompletely and subsequently cited by other authors.

**Tsunami: 28 March 1787 at ~ 12: 00 h (noon - local time)**

***Acapulco***

... the rare event that occurred yesterday [March 28, 1787] in which having left the sea its natural limits repeatedly, came with its onslaught to flood in large part the streets and houses of this port [Acapulco], [residents] have not thought since that instant in something other than saving [their] lives, leaving any answer about their businesses for the verification of the celebration of the fair, ... from the moment of the event, they were prevented by me the Governor, who tried to save their interests, removing [evacuating] them from the houses where they were in greater danger and leading them to the highest part, where in a way they were safe from the risk that threatened them; … (Vasco and de Torres, 1787). [Supplementary Fig. S2, S3].

> *... el raro suceso acaecido el día de ayer [28 de marzo, 1787] en que habiendo dejado el mar sus naturales límites repetidas veces, llegó con sus embestidas a inundar las calles y casas de este puerto en mucha parte, no han pensado desde aquel instante en otra cosa que salvar las vidas, abandonando toda contestación sobre sus comercios para el verificativo de la celebración de la feria, ...desde el instante del acaecimiento, se les*

*previno por mí el Gobernador, que procurasen salvar sus intereses, extrayéndolos de las casas donde estaban en mayor peligro y conduciéndolos a la parte más elevada, donde en cierto modo los asegurasen del riesgo que les amenazaba; …(Vasco y de Torres, 1787).*

Source: Vasco, R. y de Torres, J. J. (1787). *Documentos sobre la prolongación del término de una Feria en el Puerto de Acapulco.* Archivo General de la Nación, Instituciones Coloniales, Indiferente Virreinal, Cajas 3000-3999, Caja 3527, Expediente 024. 29 de marzo de 1787.

On March 28 around noon the sea began to retreat and raise, although without waves or particular alteration, in a way never seen before; and it became more visible at two o'clock in the afternoon, because in four minutes it went down ten feet and rise the same [2.78 m] in six [minutes], uncovering more than a hundred “varas” [83.59 m] of the beach as I observed it repeatedly, ... this was repeated every time with an increase ... at Four o'clock in the afternoon it [sea] rose more than twelve feet [3.34m] overflowing over the same pier, and entering some houses near the beach a little more than in mine, where I already considered it could continue the extraordinary reflux [Supplementary Fig.S4].

From 5 o'clock onwards, the reflux was slower, since it took a quarter of an hour for each movement, and thus the twenty-four [hours] that the element [the sea] took to recover its box [basin], with which it is peculiar and well-known (Vasco, 1787).

*El día 28 de Marzo próximo pasado como al medio día empezó a retirarse y crecer el Mar, aunque sin olas ni particular alteración, de un modo nunca visto; y a las dos de la tarde se hizo más sensible, pues en cuatro minutos bajaba diez pies [2.78m] y subía los mismos en seis, descubriéndose más de cien varas [83.59m] de Playa como lo observé repetidas veces, …esto se fue repitiendo cada vez con aumento…a las cuatro de la tarde subió más de doce pies [3.34m] rebosando por encima del mismo muelle, e introduciéndose en algunas casas cercanas a la Playa poco más que la mía, a donde ya consideraba podría llegar continuándose el extraordinario reflujo.*

*Desde las cinco en adelante fue más lento el reflujo, pues ya tardaba un cuarto de hora cada movimiento, y así pasaron siempre en disminución las veinte y cuatro que tardó el elemento en recobrar su caja, con el que le es peculiar y conocido. (Vasco, 1787).*

Source: Vasco, R. (1787) *El castellano de Acapulco participa al virrey la extraña novedad de la marea ocurrida el 28 de Marzo de 1787*. Archivo General de la Nación, Instituciones Coloniales, Indiferente Virreinal, Cajas 3000-3999, Caja 3484, Expediente 034. 2 de abril, 1787.

… An earthquake damages everything in Acapulco; the sea retreats to the rocks that are in the middle of the bay. The Philippine galleon, which was already anchored in the bay with 10 fathoms [16.7 m], was seen only with 4 fathoms [6.68 m] when the sea withdrew (du Petit-Thouars, 1840, p. 213)

*…un temblor de tierra arruina todo Acapulco; el mar se retira hasta las rocas que están en la mitad de la bahía. El galeón de Filipinas, que ya estaba fondeado en la rada con 10 brazas [16.7 m], se vio solo con 4 brazas [6.68 m] cuando el mar se retiró* (du Petit-Thouars, 1840, p. 213)

Source: du Petit-Thouars, A. (1841). *Voyage autour du monde sur la frégate "la Vénus" pendant les années 1836-1839*. Vol II. Paris, Gide.

*On the 14th of March 1787, the whole town was ruined. The sea withdrew, leaving the rocks of the Punta Manzanilla (in the town bay) dry. The Philippine, Nao, was anchored at the time in the port, and was left in four fathom; before the tide returned, — showing a fall of thirty-six feet* [10 m]. (Belcher, 1843; p.148).

Source: Belcher, E. (1843). *Narrative of a voyage round the world: performed in Her Majesty's ship Sulphur, during the years 1836-1842, including details of the naval operations in China, from Dec. 1840 to Nov. 1841.* London: H. Colburn

On 28th of the same month at twelve o'clock at daytime the sea withdrew in Acapulco, and began to rise: this movement was increased at two in the afternoon because in four minutes it went down 10 feet, and rose another many [feet] in 6 [minutes], overflowing the [sea] water above the pier; all this was accompanied by strong earth tremors, which ceased after twenty-four hours that it took the sea to recover its old box [basin].

On the open beach, and where it [the sea] did not find the obstacles of mountains like in Acapulco, the sea leaving its basin, drowned a multitude of cattle that grazed in the pastures: the butler of the estate D. Francisco Rivas, Regidor of Oaxaca, seeing it [the sea] coming, he climbed into a big tree where he saved his life, very afraid that it [the sea] would eat the roots, and [the tree would] come down; but after it [the sea] retired to its centre, he [the butler] advanced inland on foot, since the horse he rode perished among the waves ... … (Cavo y Bustamante, 1838: pp. 68-69).

> *En 28 del mismo mes a las doce del día se retiró el mar en Acapulco, y comenzó a crecer: este movimiento se aumentó a las dos de la tarde pues en cuatro minutos bajaba 10 pies, y subía otro tantos en 6, rebosando las aguas por sobre el muelle; todo esto fue acompañado de fuertes temblores de tierra, que cesaron a las veinte y cuatro horas que tardo el mar en recobrar su antigua caja.*
>
> *En la Playa abierta, y en que no encontró los obstáculos de montañas que en Acapulco, saliendo de caja el mar, ahogó multitud de ganado mayor que pastaba en las dehesas: el mayordomo de la hacienda de D. Francisco Rivas, Regidor de Oaxaca, viéndolo venir, se trepó en un árbol corpulento donde salvó la vida, temerosísimo de que comidas las raíces, viniese abajo; pero luego que se retiró a su centro, avanzó tierra adentro a pie, pues el caballo que montaba pereció entre las olas ...* (Cavo y Bustamante, 1838: pp. 68-69).

Source: Cavo, A., Bustamante, C. María de. (1838). *Suplemento a la historia de los tres siglos de México, durante el gobierno español.* México: C.M. de Bustamante

*Igualapa*

Don Francisco Gutiérrez de Terán, Mayor of Igualapan ... says that at the time of the first [earthquake], being several unhappy [fishermen] of those fisheries making bars to catch fish, and already some mounted on horseback after having collected and put [fish] in their nets, they saw with astonishment the sea to withdraw more than one league [4.19 km][9], discovering lands of different colours, rocks and trees [coral], and that with the same speed it [the sea] fled from his sight, it returned again and several times, leaving thousands of fish in the district that was left without water, and in front of them it [the sea] made many pieces to eleven of them [fishermen], leaving them hanging and stuck between the sticks [trees] of a mountain, which is like a league and a half [8.38 km] from the sea, and of excessive height, escaping only some, although very wounded and injured, which are the ones that have referred to the case, all neighbours of that jurisdiction. (Gaceta de México, 1787b; pp. 341-342). [Supplementary Fig. S5].

> *Don Francisco Gutiérrez de Terán, Alcalde de Igualapan…dice que al tiempo del primero [sismo], estando varios infelices de aquellas pesquerías haciendo barras para coger pescado, y ya algunos montados a caballo después de haberlo recogido y metido en sus redes, vieron con asombro retirarse el mar más de una legua, descubriéndose tierras de diversos colores, peñascos y árboles, y que con la misma velocidad se huyó de su vista, volvió otra vez y otras, dejando millares de pescados en el distrito que quedó sin agua, y antecogiéndolos hizo muchos pedazos a once de ellos, dejándolos colgados y metidos entre los palos de un monte, que dista como una legua y media del mar, y de excesiva altura, escapando solamente algunos, aunque muy maltratados y heridos, que son los que han referido el caso, todos vecinos de aquella jurisdicción. (Gaceta de México, 1787b; pp. 341-342)*

Source: Gaceta de México (1787b). Tomo II, Num. 34, martes 1 de mayo de 1787.

*Jamiltepec*

... some coastal people could save their lives up in the trees until the waters receded (Gay, 1838; p. 346).

> *...algunos costeños pudieron salvar sus vidas subidos en los árboles hasta que se retiraron las aguas (Gay, 1881; p. 346).*

Source: Gay, J. A. (1881) *Historia de Oaxaca*. Tomo II. México, Imprenta del Comercio de Dublan y Cia.

*Alotengo*

Some fishermen, at the Alotengo bar, at eleven o'clock that day, saw with astonishment that the sea was retreating, leaving uncovered lands of different colours, cliffs and underwater trees [corals] in more than one league of extension, and then retreating with the speed with which it had moved away, it covered with its waves the forests of the beach, in which it move inland more than two leagues, leaving among the branches of the trees, when returning to its box [basin], many and varied dead fish; some fishermen perished and others were saved very injured (Gay, 1881; p. 346). [Supplementary Fig. S5].

> *Algunos pescadores, en la barra de Alotengo, a las once horas de ese día, vieron con asombro que el mar se retiraba, dejando descubiertas en más de una legua de extensión, tierras de diversos colores, peñascos y árboles submarinos, y que retrocediendo luego con la velocidad con que se había alejado, cubría con sus ondas los bosques de la playa, en que se internó más de dos leguas, dejando entre las ramas de los árboles, al volver a su caja, muchos y variados peces muertos; algunos pescadores perecieron y otros pudieron salvarse muy estropeados.* (Gay, 1881; p. 346).

Source: Gay, J. A. (1881) *Historia de Oaxaca*. Tomo II. México, Imprenta del Comercio de Dublan y Cia.

**Tsunami: Tuesday 3 April 1787**

*Tehuantepec*

Lieutenant Colonel Don Tomás de Mollinedo, Mayor of this Jurisdiction [Tehuantepec], participates ... that on April 3 at ten o'clock in the morning another [earthquake] repeated for some ten or twelve minutes with such violence...
He also participates that in the places near the sea, distant four leagues [16.76 km] from the Villa, the movements have been felt with a strange commotion of the [sea] waters, and frightening roar of that one [sea], resulting in throwing fish of extraordinary beauty and shells never seen on the beach ... (Gaceta de México, 1787b; pp. 342-343).

> *El Teniente Coronel Don Tomás de Mollinedo, Alcalde mayor de esta Jurisdicción [Tehuantepec], participa… que el día 3 de abril como a las diez de la mañana repitió otro [sismo] como por diez o doce minutos con tanta violencia…*
> *Igualmente participa que en los lugares cercanos al mar distantes cuatro leguas de la Villa se han sentido los movimientos con una extrañísima conmoción de las aguas, y espantoso bramido de aquél, resultando arrojar a la playa peces de extraordinario grandor y conchas nunca vistas, …* (Gaceta de México, 1787b; pp. 342-343).

Source: Gaceta de México (1787b). Tomo II, Num. 34, martes 1 de mayo de 1787.

*Litoral Pochula-Juquila*

...resulting in throwing fish of extraordinary beauty and shells never seen on the coast, whose magnitude has been admired by all. These same phenomena were verified on the coast of Pochula and Juquila (Orozco y Berra, 1888; p. 344).

*...resultando arrojar a la costa peces de extraordinario grandor y conchas nunca vistas cuya magnitud se ha hecho admirar de todos. Estos mismos fenómenos se verificaron en el litoral de Pochula y Juquila.* (Orozco y Berra, 1888; p. 344).

Source: Orozco y Berra, J. (1888). Efemérides Sísmicas Mexicanas. Memorias de la Sociedad Cientírifica "Antonio Alzate*" I*(8), 303–541.

**Tsunamis produced by the 1787 earthquakes**

Mexico, near San Marcos [Guerrero]. [Earthquakes] Felt along coast of Oaxaca, Michoacán, and south of Tehuantepec. Sea withdrew, leaving rocks of Punta Manzanilla [*sic*] dry, and returned very high, estimated at 36 feet by vessel at anchor (Heck, 1947).

*Mexico, near San Marcos. Felt along coast of Oaxaca, Michoacán, and south of Tehuantepec. Sea retired, leaving rocks of Punta Manzanilla [sic] dry, and returned very high, estimated at 36 feet by vessel at anchor* (Heck, 1947).

Source: Heck, N. (1947). List of Seismic Sea Waves. *Bulletin of the Seismological Society of America,* 37(4), 269-286.

**Supplementary Figures**

Supplementary Figure S2. Acapulco Port map, 1791. Drafted by Corvetas de S. M. *Descubierta y Atrevida*. Scale 1 nautical mile = 15cm. Bibliotheque Nacional de France. Please see figure at: https://gallica.bnf.fr/ark:/12148/btv1b531233366 . Last access online: 7/sep./2019.

Source: Plano del Puerto de Acapulco, 1791. Levantado por las Corvetas de S. M. Descubierta y Atrevida. Escala 1 milla marítima= 15cm. Bibliotheque Nacional de France. Available at: https://gallica.bnf.fr/ark:/12148/btv1b531233366.

Supplemental Figure S3. Map of the bay, port and castle of San Diego de Acapulco, 1712. Please see Figure at: http://pares.mcu.es/ParesBusquedas20/catalogo/show/20932 . AGI, ES.41091.AGI/27.17//MP-MEXICO,106. Key: El Castillo de San Diego: 1. El rebellin, 2.Puente levadizo, 3.El Foso, 4. La Capilla, 5. Rancho Real Santa Bárbara, 6. Casa mata de bajo de tierra, 7. Los cuarteles, 8. Casa matas, 9.Casa de balas y cuerdas, 10. La garita, 11. Casa del Castellano, 12. Los cuarteles, 13. Sala de armas, 14. Quespe de Guardia, 15.El tanque. Bahía: A. Castillo de San Diego, B. La Catedral, C. Casa del Castellano, D. Hospital, E. San Nicolás, G. La Contaduría, H. San Joseph, I. La Feria, K. El Marqués, L. La Boca Chica, M. Isla de Chinos, N. El Farallón, O. Punta del Grifo, P. La Boca Grande, Q. Playa Honda.

Source: Mapa de la bahía, puerto y castillo de San Diego de Acapulco, 1712. Archivo General de Indias, Sevilla, España. ES.41091.AGI/27.17//MP-MEXICO,106.

Supplemental Figure S4. Map of the Acapulco Port, 1889, with longitudinal profile.

Please see Figure at: http://132.248.9.195/imp_mzo_2011/CG%20Guerrero/CGGRO-V5-32-CGE-7271-A.pdf . Antonio Pintos, Presidente Municipal de Acapulco (Lallier, E., 1889).

Source: Lallier, E. (1889) Plano Topografico de la Ciudad y Puerto de Acapulco Levantado y Ejecutado por el Ingeniero Enrique Lallier según el concepto y con la protección de los Señores Francisco Leyva Prefecto Político del Distrito y Antonio Pintos Presidente Municipal de Acapulco 1889. Impreso Britton & Rey. Lith. S.F. Clasificación Local: CGGRO-V5-32-CGE-7271-A. Access on MAPAMEX. Availabe at: http://132.248.9.195/imp_mzo_2011/CG%20Guerrero/CGGRO-V5-32-CGE-7271-A.pdf last access: 8/Nov/2019

Supplemental Figure S5. A part of the Obispado de Oaxaca Map (1824).

Please see Figure at: http://132.248.9.195/imp_nov_2010/oyboax/1150-20.pdf Red circles - Igualalpa and Xamiltepec villages, black square – Ometepec, and blue circle - Laguna de Alotengo (Peláez, 1824), currently named Laguna Corralero.

Source: Peláez, L. (1824). Descripcion Geografica de la Intendencia y Obispado de Oaxaca. Año de 1824. Clasificación Local: 1150-OYB-7272-A Biblioteca Orozco y Berra. Access on MAPAMEX. Digital file available at: http://132.248.9.195/imp_nov_2010/oyboax/1150-20.pdf last access: 8/Nov/2019.

**References**


1. Gaceta de México. Tomo II, Num. 32, martes 17 de abril de 1787. (1787).
2. Gaceta de México. Tomo II, Num. 34, martes 1 de mayo de 1787. (1787).
3. Suarez, G. & Albini, P. Evidence for Great Tsunamigenic Earthquakes (M 8.6) along the Mexican Subduction Zone. *B. Seismol. Soc. Am.* **99**, 892–896, https://doi.org/10.1785/0120080201 (2009).
4. National Geophysical Data Center/World Data Service. NCEI/WDS Global Historical Tsunami Database, https://doi.org/10.7289/v5pn93h7 (2019).
5. Orozco y Berra, J. Efemérides Sísmicas Mexicanas. *Memorias de la Sociedad Científica 'Antonio Alzate'* **I**, 303–541 (1888).
6. Sánchez Devora, A. J. & Farreras Sanz, S. *Catalog of tsunamis on the western coast of Mexico. Report SE-50*. (World Data Center A for Solid Earth Geophysics, NOAA, National Geophysical Data Center, Boulder, Colorado, 1993).
7. Heck, N. H. List of seismic sea waves. *Bulletin of the Seismological Society of America* **37**, 269–286 (1947).
8. Soloviev, S. L. & Go, C. N. *A catalogue of tsunamis on the eastern shore of the Pacific Ocean (1513-1968)*. (Nauka Publishing House. Can. Transi. Fish. Aquat., 5078, 1984, 1975).
9. Vasco, R. & de Torres, J. J. *Documentos sobre la prolongación del término de una Feria en el Puerto de Acapulco a petición de los comisarios del comercio de Filipinas por haberse visto entorpecido debido a anomalías en la marea*. 29 de marazo de 1787. Archivo General de la Nación, Instituciones Coloniales, Indiferente Virreinal, Cajas 3000-3999, Caja 3527, Expediente 024 (1787).
10. Vasco, R. *El castellano de Acapulco participa al virrey la extraña novedad de la marea ocurrida el 28 de Marzo de 1787*. Archivo General de la Nación, Instituciones Coloniales, Indiferente Virreinal, Cajas 3000-3999, Caja 3484, Expediente 034 (1787).
11. du Petit-Thouars, A. *Voyage autour du monde sur la frégate 'la Vénus' pendant les années 1836-1839*. vol. III (Gide, 1841).

12. Belcher, E. *Narrative of a voyage round the world: performed in Her Majesty's ship Sulphur, during the years 1836-1842, including details of the naval operations in China, from Dec. 1840 to Nov. 1841*. (H. Colburn, 1843).

13. Cavo, A. & Bustamante, C. M. de. *Suplemento a la historia de los tres siglos de México, durante el gobierno español*. vol. III (Imprenta de la Testamentaria de D. Alejandro Valdes, 1836).

14. Gay, J. A. *Historia de Oaxaca*. Vol. Tomo II (Imprenta del Comercio de Dublan y Cia., 1881).

**Supplemental Figure S6. Grain size statistics**

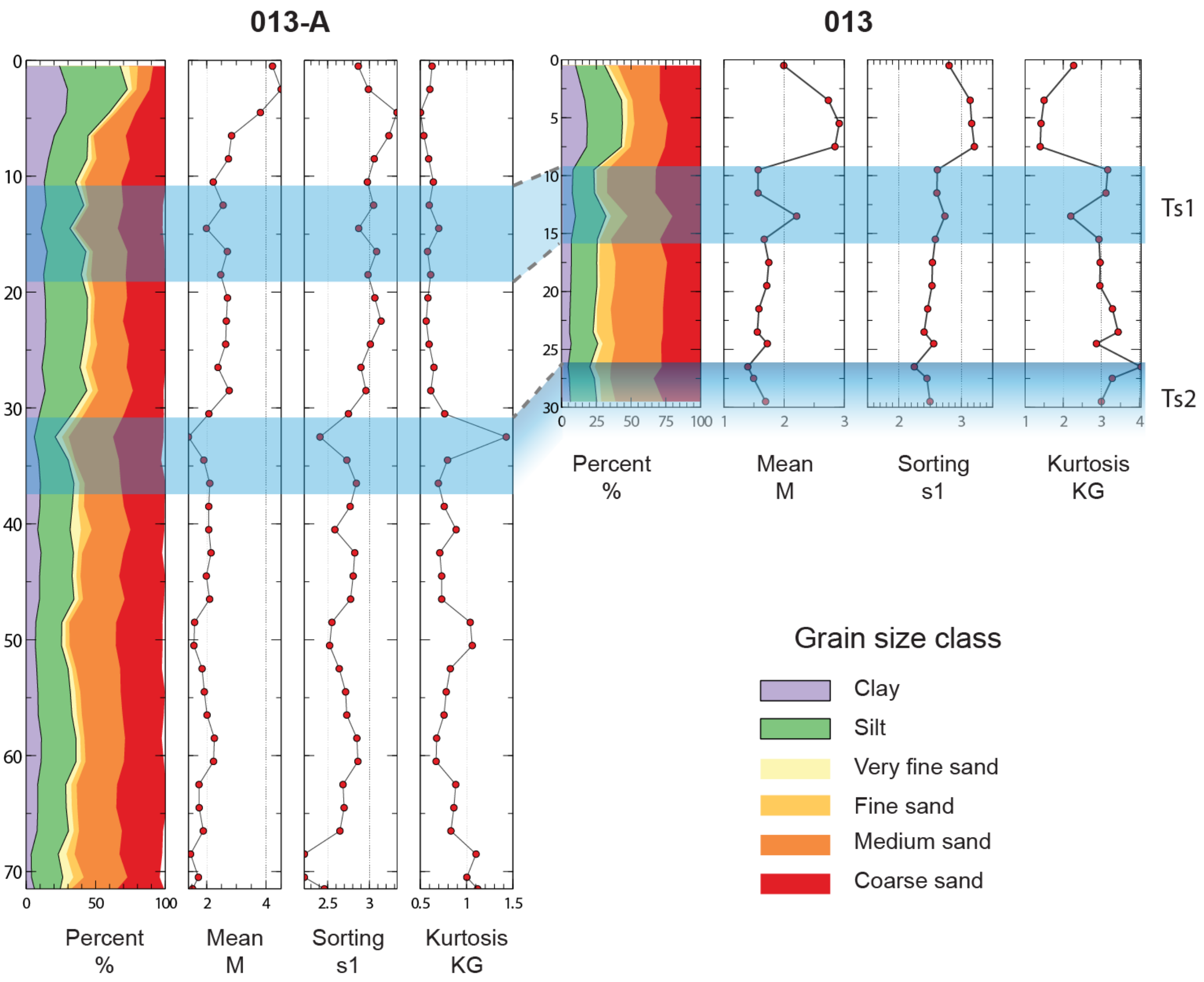


Supplemental Figure S6. Grain size statistics of 013-A and 013. Tsunami deposits (TS1 and TS2) are marked with a blue band. Statistics were calculated using the GRADISTAT[1] program applying Logarithmic graphical measures[2].

**References**


1. Blott, S. J. & Pye, K. GRADISTAT: a grain size distribution and statistics package for the analysis of unconsolidated sediments. *Earth Surface Processes and Landforms* **26**, 1237–1248, https://doi.org/10.1002/esp.261 (2001).

2. Folk, R. L. & Ward, W. C. Brazos River Bar – a study in the significance of grain size parameters. *Journal of Sedimentary Petrology* **27**, 3–26 (1957).

**Supplementary Information. Microfossil data**

Supplementary Table S2. Sampling depths for microfossil analysis.

| Sample Number | Sample depth (cm) | Sample Number | Sample depth (cm) |
|---|---|---|---|
| 1 | 0-1 | 13 | 30-31 |
| 2 | 4-5 | 14 | 34-35 |
| 3 | 6-7 | 15 | 36-37 |
| 4 | 8-9 | 16 | 39-40 |
| 5 | 10-11 | 17 | 42-43 |
| 6 | 12-13 | 18 | 44-45 |
| 7 | 14-15 | 19 | 50-51 |
| 8 | 16-17 | 20 | 53-54 |
| 9 | 18-19 | 21 | 59-60 |
| 10 | 20-21 | 22 | 63-64 |
| 11 | 22-23 | 23 | 69-70 |
| 12 | 26-27 | | |

Supplementary Figure S7. Diatom and Dinoflagellates assemblages

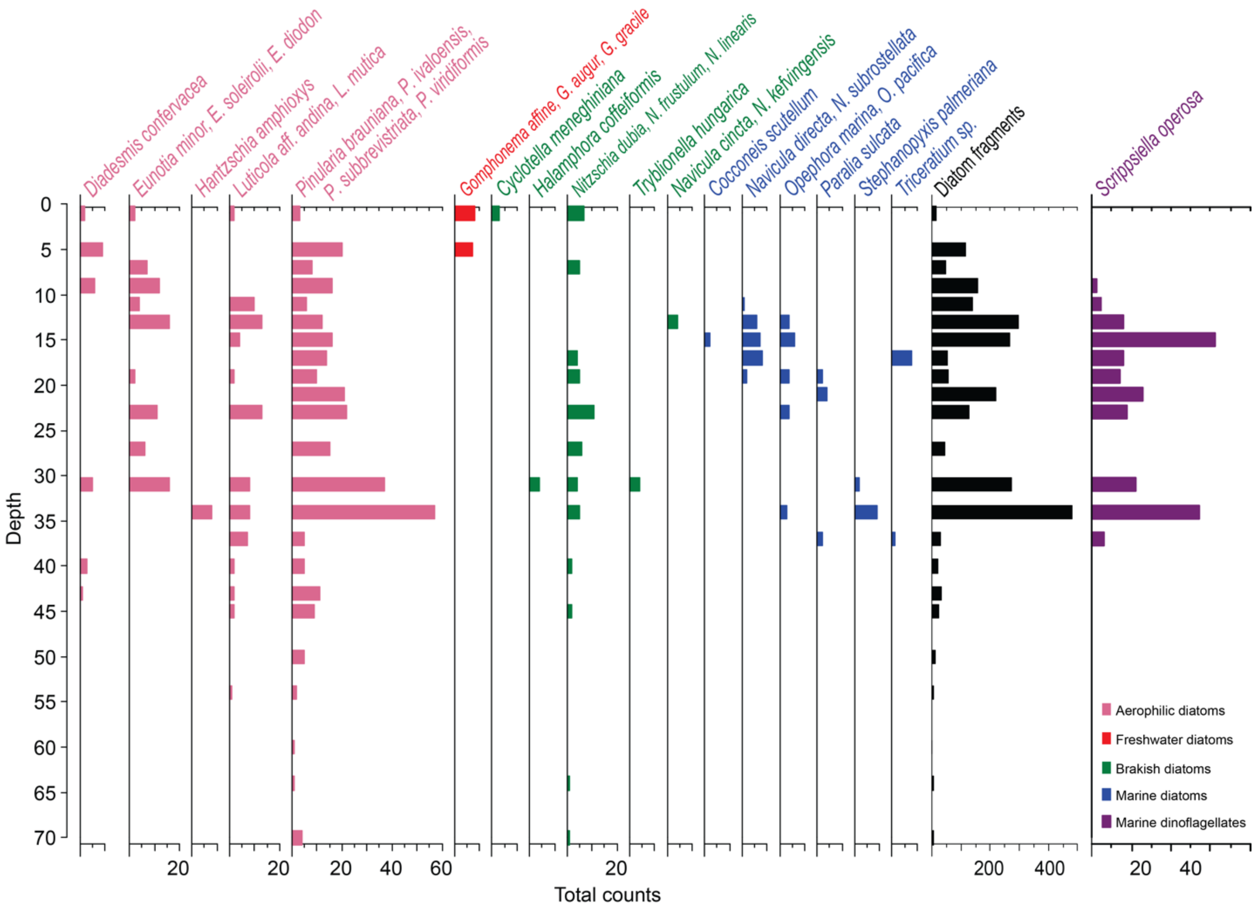

References

1. Bąk, M.,*et al. Klucz do oznaczania okrzemek w fitobentosie na potrzeby oceny stanu ekologicznego wód powierzchniowych w Polsce*. Główny Inspektorat Ochrony Środowiska, Warszawa. (2012).
2. Caljon, A. *Brackish-water phytoplankton of the Flemish lowland*. Dr W. Jung Publishers, The Hague, Netherlands, https://doi.org/10.1007/978-94-009-6554-6 (1984).
3. Cantonati, M. & Lange Bertalot, H. Diatom monitors of close-to-pristine, very-low alkalinity habitats - three new Eunotia species from springs in Nature Parks of the south-eastern Alps, *Journal of Limnology,* **70**, 209-221, https://doi.org/10.4081/jlimnol.2011.209 (2011).
4. Krammer, K. *Diatoms of Europe. The Genus Pinnularia.* A.R.G. Gantner Verlag. Kommanditgesellschaft, Königstein, Vol. 1: 703 pages. (2000).
5. Hernández-Becerril, D. U. & Bravo-Sierra, E. New records of planktonic dinoflagellates (Dinophyceae) from the Mexican Pacific Ocean. *Botanica Marina* **47**, 417–423, https://doi.org/10.1515/BOT.2004.051 (2004).
6. Lange-Bertalot, H. *Iconographia Diatomologica*, Vol. 5. Koeltz Scientific Books, 695 p. (1998).
7. Moreno, J. L., Licea, S. & Santoyo, H. *Diatomeas del Golfo de California*. Universidad Autónoma de Baja California Sur, 272 p. (1996).
8. Siqueiros Beltrones, D. A. *Diatomeas bentónicas de la Península de Baja California: diversidad y potencial ecológico.* Instituto Politécnico Nacional y Universidad Autónoma de Baja California Sur, 102 pag. (2002).
9. Taylor, J. C., Harding, W. R. & Archibald C.G.M. *An Illustrated Guide to Some Common Diatom Species from South Africa*. Report to the Water Research Commission. (2007).
10. Wehr, J.D., Sheath, R.G. & Kociolek, J.P. *Freshwater Algae of North America: Ecology and Classification* (Second Edition). Elsevier Inc. 1050 p., https://doi.org/10.1016/C2010-0-66664-8 (2015).
11. Witkowski, A., Lange-Bertalot, H. and Metzeltin, D. *Diatom flora of marine coasts I. Iconographia Diatomologica* **7**, 925 p. (2000).

**Supplemental Figure S8. Elemental composition ratios**

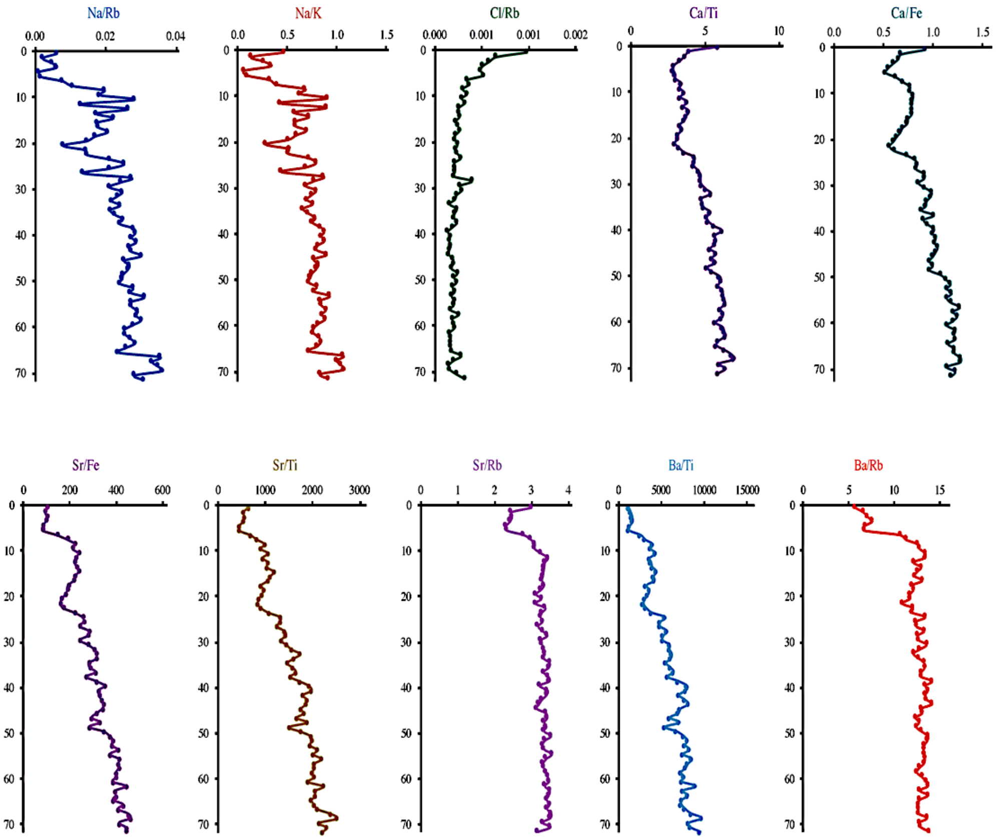


Supplemental Figure S8. Elemental composition of pit 013 units shows relatively higher content of Na/Rb, Na/K, Ca/Ti, Ca/Fe, Sr/Fe, Sr/Ti, Ba/Ti, Ba/Rb ratios indicative of marine influence (vs. terrestrial source).

**Supplementary Information - Stochastic slip and associated coseismic deformation models**

We include below some examples of the generated 600 stochastic heterogenous slip models in the magnitude range M8.0 to M8.8 using the method described by Melgar et al.[1] and of the calculated vertical deformation using known analytical solutions[2].

A.

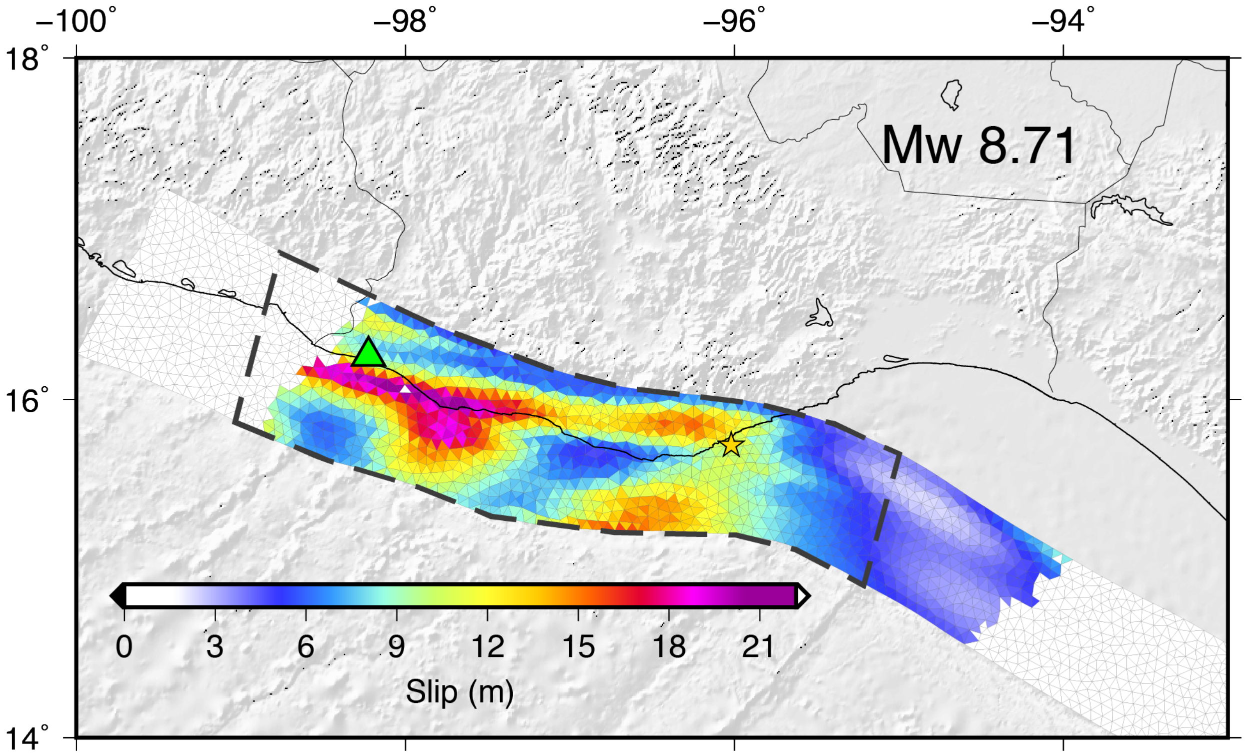


B.

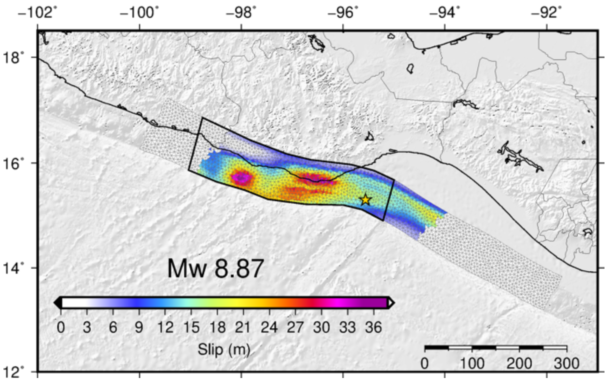


C.

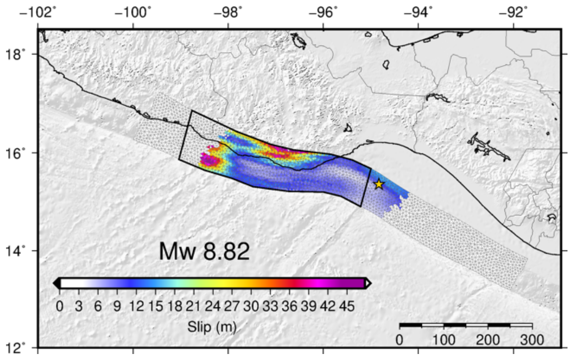

D.

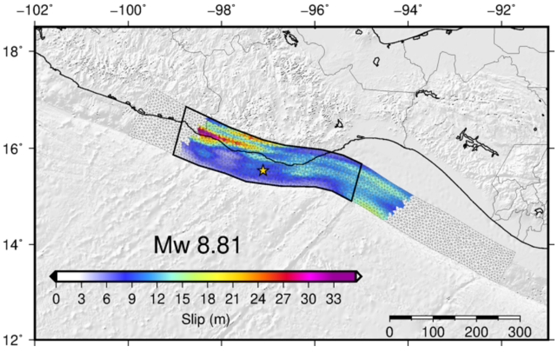


E.

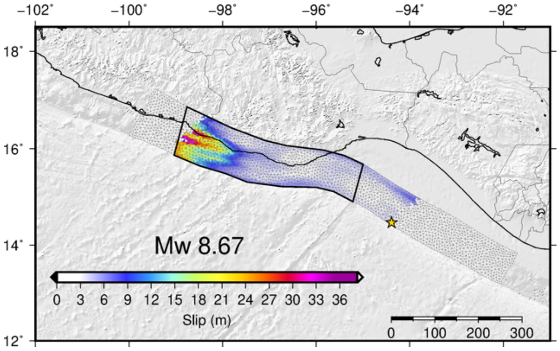


Supplementary Figure S9. Examples of stochastic heterogeneous slip models. A. Green triangle – Corralero site, dashed line – rupture area by Suarez and Albini[3] matched with the slab model; B - E. Examples of 600 runs for stochastic heterogenous slip models.

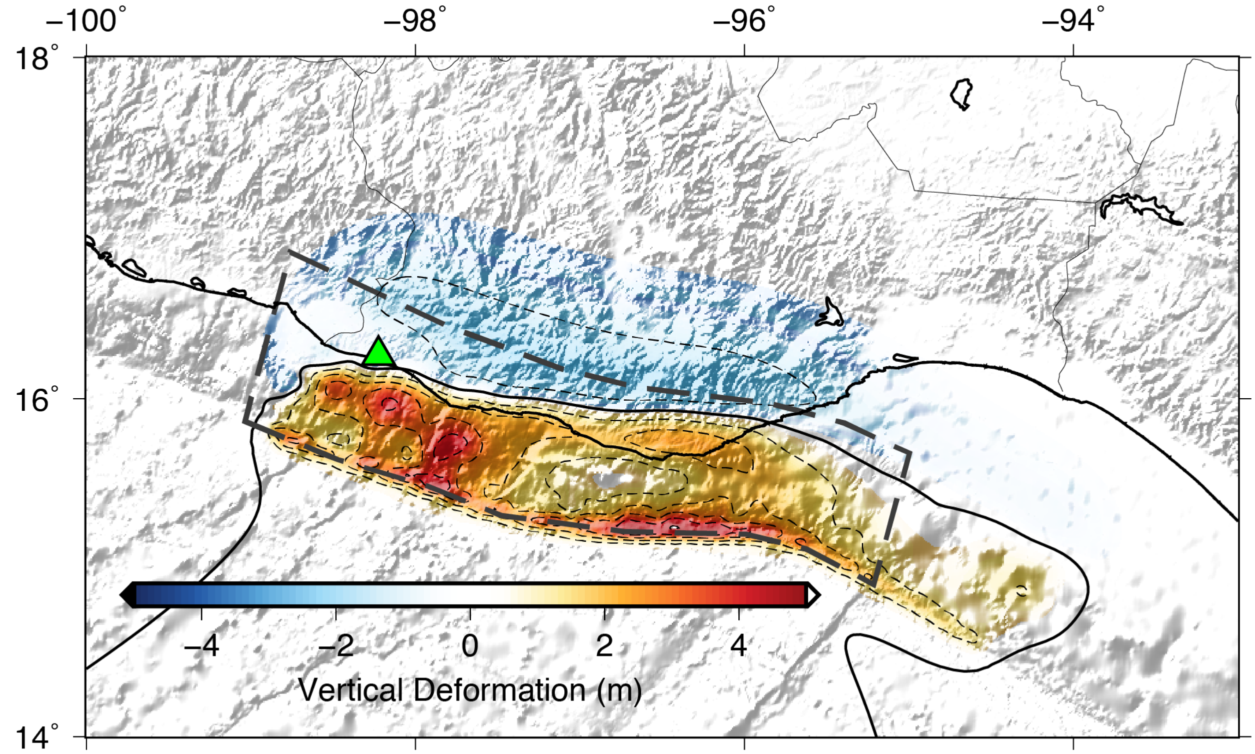


Supplementary Figure S10. Coseismic vertical deformation by the M8.6 earthquake[3] in 1787. Contour lines show deformation every 1 m, except for the 0 m continuous contour line. Dashed line – rupture zone after Suarez and Albini[3].

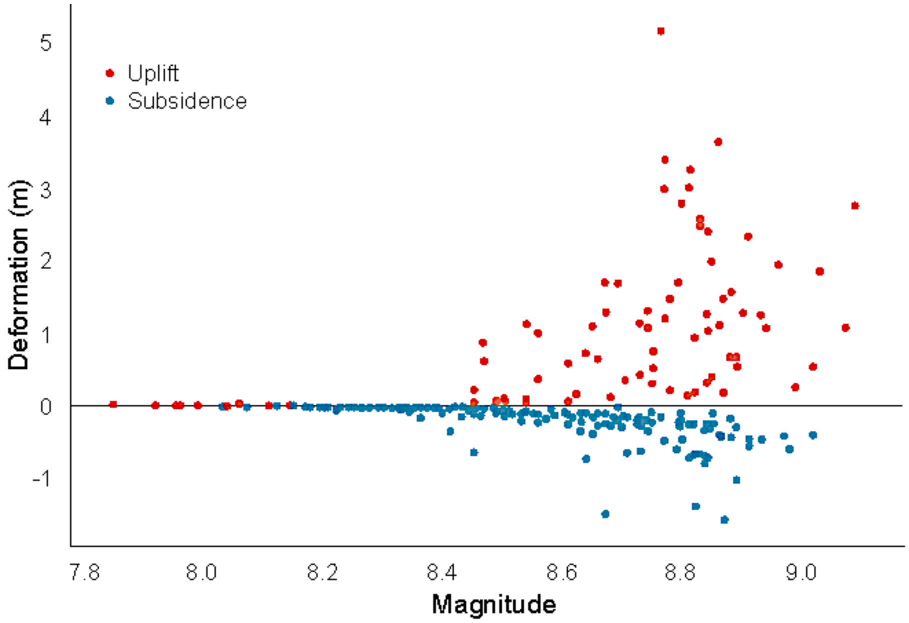


Supplementary Figure S11. Coseismic vertical deformation based on 600 stochastic slip models in a broad magnitude range within the expected rupture region. The magnitude range M8.5-M8.7 and 65% of the models show co-seismic subsidence.

**References**

1. Melgar, D., LeVeque, R. J., Dreger, D. S. & Allen, R. M. Kinematic rupture scenarios and synthetic displacement data: An example application to the Cascadia subduction zone: Rupture Scenarios and Synthetic Data. *J. Geophys. Res. -Sol. Ea.* **121**, 6658–6674, https://doi.org/10.1002/2016JB013314 (2016).

2. Okada, Y. Surface deformation due to shear and tensile faults in a half-space. *B. Seismol. Soc. Am.* **75**, 1135–1154 (1985).

3. Suarez, G. & Albini, P. Evidence for Great Tsunamigenic Earthquakes (M 8.6) along the Mexican Subduction Zone. *B. Seismol. Soc. Am.* **99**, 892–896, https://doi.org/10.1785/0120080201 (2009).

**Supplementary Information 6. Acknowledgements**

We wish to acknowledge participants of other surveys where evidence have been mostly obliterated by agriculture and salt extraction activities:

Gerardo Suárez helped in preliminary search in the field during which no tsunami deposits where identified.

Karen Chang and Omar Ruiz helped in the field and worked on parallel topics for their graduate thesis supervised by MTR.

Eder Arellano Rodriguez from the Corralero, Oaxaca, community for help with digging multiple pits and coring during all the field surveys.

Corralero, Oaxaca, community for kindly sharing their memories and tsunami perception, and for an always friendly welcoming to their community.

N.C. thanks tsunami modelling feedback by D. Sugawara.
